# The antioxidant and antimicrobial activity of ethanolic extract in roots, stems, and leaves of three commercial *Cymbopogon* species

**DOI:** 10.1186/s12906-024-04573-4

**Published:** 2024-07-18

**Authors:** Dwi Kusuma Wahyuni, Viol Dhea Kharisma, Ahmad Affan Ali Murtadlo, Cici Tya Rahmawati, Alvi Jauharotus Syukriya, Sehanat Prasongsuk, Sreeramanan Subramaniam, Anjar Tri Wibowo, Hery Purnobasuki

**Affiliations:** 1 Department of Biology, Faculty of Science and Technology, Universitas Airlangga Surabaya, 60115 East Java, Indonesia; 2 Program in Biotechnology, Faculty of Science, Chulalongkorn University, 10330 Bangkok, Thailand; 3 Plant Biomass Utilization Research Unit, Department of Botany, Faculty of Science, Chulalongkorn University, 10330 Bangkok, Thailand; 4 School of Biological Science, Universiti Sains Malaysia, 11800 Georgetown, Malaysia

**Keywords:** Antimicrobial resistance treatment, Antioxidant, *Candida albicans*, *Bacillus subtilis*, *Staphylococcus aureus*, *Escherichia coli*, *Cymbopogon* spp., Molecular docking, Biological pathway

## Abstract

**Background:**

*Cymbopogon* is a member of the family Poaceae and has been explored for its phytochemicals and bioactivities. Although the antimicrobial activities of *Cymbopogon* spp*.* extracts have been extensively studied, comprehensive analyses are required to identify promising compounds for the treatment of antimicrobial resistance. Therefore, this study investigated the antioxidant and antimicrobial properties of *Cymbopogon spp.* ethanolic extracts in every single organ.

**Methods:**

Ethanolic extracts were obtained from three Indonesian commercial species of *Cymbopogon spp.*, namely *Cymbopogon citratus* (L.) Rendle*, Cymbopogon nardus* (DC.) Spatf., and *Cymbopogon winterianus* Jowitt. The leaf, stem, and root extracts were evaluated via metabolite profiling using gas chromatography-mass spectrometry (GC–MS). In silico and in vitro analyses were used to evaluate the antioxidant and antimicrobial properties of the *Cymbopogon* spp. ethanolic extracts. In addition, bioactivity was measured using cytotoxicity assays. Antioxidant assays were performed using 1,1-diphenyl-2-picrylhydrazyl (DPPH) and 2,2-azino-bis [3-ethylbenzothiazoline-6-sulfonic acid (ABTS) to determine toxicity to Huh7it-1 cells using a tetrazolium bromide (MTT) assay. Finally, the antimicrobial activity of these extracts was evaluated against *Candida albicans*, *Bacillus subtilis*, *Staphylococcus aureus*, and *Escherichia coli* using a well diffusion assay.

**Results:**

GC–MS analysis revealed 53 metabolites. Of these, 2,5-bis(1,1-dimethylethyl)- phenol (27.87%), alpha-cadinol (26.76%), and 1,2-dimethoxy-4-(1-propenyl)-benzene (20.56%) were the predominant compounds. *C. winterianus* and *C. nardus* leaves exhibited the highest antioxidant activity against DPPH and ABTS, respectively. Contrastingly, the MTT assay showed low cytotoxicity. *C. nardus* leaf extract exhibited the highest antimicrobial activity against *E. coli* and *S. aureus,* whereas *C. winterianus* stem extract showed the highest activity against *B. substilis*. Furthermore, computational pathway analysis predicted that antimicrobial activity mechanisms were related to antioxidant activity.

**Conclusions:**

These findings demonstrate that the leaves had strong antioxidant activity, whereas both the leaves and stems showed great antimicrobial activity. Furthermore, all *Cymbopogon* spp. ethanolic extracts showed low toxicity. These findings provide a foundation for future studies that assess the clinical safety of *Cymbopogon* spp. as novel drug candidates.

**Supplementary Information:**

The online version contains supplementary material available at 10.1186/s12906-024-04573-4.

## Background

Infections are a serious global health problem [1]. In Indonesia, 28.1% of the primary causes of death are infections. Infection is caused by various microorganisms, such as bacteria, viruses, fungi, and protozoa [2]. The World Health Organization (WHO) has issued a list of priority pathogens to enhance research efforts in the search for new antibiotics that overcome drug resistance [3]. This necessitates the identification of novel sources of antimicrobial drugs, such as plant-derived natural products [4, 5].

Approximately 80% of the world population uses natural compounds derived from medicinal plants [1]. However, many potentially medicinal plants in Indonesia have not been studied. Of the 9,600 plant species with medicinal properties, only 700–1000 are used for medicinal purposes. One of these species is serai (*Cymbopogon* spp.), which has numerous uses in Indonesia, including as a spice, drink, and source of essential oils. *Cymbopogon* spp.*,* which belongs to the family Poaceae, consists of various species, including seasoned lemongrass (*Cymbopogon citratus* (L.) Rendle) and fragrant lemongrass (citronella; *Cymbopogon nardus* (DC.) Spatf and *Cymbopogon winterianus* Jowitt) [6, 7].

The bioactivity of *Cymbopogon* spp. has been explored extensively. The antimicrobial activity of *C. flexuoxus* [8], *C. citratus* [9–12], *C. nardus* [13, 14], and *C. schoenanthus* [15], has been studied. Moreover, the pesticidal activity of *C. citratus* [16], and *C. winterianus* [17], as well as the antioxidant activity of *C. citratus* [18, 19], have been investigated. The essential oil of *Cymbopogon* spp. plays an important role in some lemongrass bioactivities [8]. This essential oil has shown excellent biocompatibility and few side effects in human studies [20]. Furthermore, the molecular mechanisms of the bioactive compounds of *Cymbopogon* spp. against some microbes have been evaluated using proteomic analyses [8].

Wahyuni et al., [21] elaborated that metabolite profiles and bioactivities differed with each plant organ. Similarly, antimicrobial and antioxidant activity assays of *Cymbopogon* spp*.* organs and computational prediction of the mechanism pathway have been conducted in this study, as there are few reports about antimicrobial and antioxidant activity assays of organ/plant part of *Cymbopogon* spp*.* and the mechanism pathway prediction computationally. Moreover, in this study, ethanol was used as the solvent because its structure enables the dissolution of polar compounds such as water, non-polar compounds, hydrophilic compounds such as hexane, and hydrophobic compounds. Ethanol is also used as a medicinal solvent owing to its low toxicity and nonpolar capabilities. Similarly, ethanol extract is more soluble [22].

This study aimed to explore and compare the antioxidant and antimicrobial activities of roots, stems, and leaves derived from three commercial Indonesian *Cymbopogon* spp., as well as the mechanisms of inhibition. Our findings could enable the pharmaceutical applications of these three *Cymbopogon* spp. in Indonesia.

## Methods

### Plant material collection and identification

The three commercials Indonesian *Cymbopogon* spp. used in this study were *Cymbopogon citratus* (DC.) (Stapf)*, Cymbopogon nardus* (L.) Rendl*,* and *Cymbopogon winterianus* Jowitt. The plants were obtained from the Medicinal Plant Garden (Taman Husada Graha Famili) in Surabaya, East Java, Indonesia (7º18′12.2’’S 112º41′12.7’’7E). Samples were collected and authenticated by the authors in the Plant Systematics Laboratory, Department of Biology, Faculty of Science and Technology, Universitas Airlangga. A voucher specimen was deposited to the Plant Systematics Laboratory, Department of Biology, Faculty of Science and Technology, Universitas Airlangga (No. CC.0110292022; CN.0110292022; and CW.0110292022).

## Extraction

The leaves, stems, and roots of *Cymbopogon* spp. were air-dried and ground into a powder (20 mesh size). Next, 10 g of the ground samples were macerated sequentially in absolute ethanol (pro analysis; Merck, Darmstadt, Germany) at a ratio of 1:10. Each maceration was done thrice for 24 h at room temperature (28 ± 2 °C). The resulting extracts were filtered through filter paper, evaporated on a rotary evaporator at 60ºC to acquire a dry residue, weighed to calculate the yield of each extract, and stored at 4ºC.

## Metabolite profiling by gas chromatography-mass spectrometry (GC–MS)

GC–MS was used to establish compound profiles from the ethanolic extracts of *Cymbopogon* spp. GC–MS analysis was performed using an Agilent GC-MSD (19091S-433UI; Agilent Technologies, Santa Clara, CA, United States) equipped with a capillary column (30 m × 250 µm × 0.25 µm), and a mass detector was operated in electron impact mode with full scan (50,550 amu). Helium was used as the carrier gas at a flow rate of 3 mL/min and a total flow rate of 14 mL/min. The injector was operated at 280 °C, whereas the oven temperature was programmed at an initial temperature of 60 °C and final temperature of 250 °C. Peaks in the chromatograms were identified based on their mass spectra. The compounds were identified through comparisons of their mass spectra with those in the Standard Reference Database (version 02. L; National Institute of Standards and Technology, Gaithersburg, MD, USA). Compounds with similarities > 80% were used in this study. The relative percentage of each component was calculated as the relative percentage of the total peak area in the chromatograph.

## In silico analysis of antimicrobial and antioxidant pathways

### Ligand retrieval

Compounds from *Cymbopogon* spp. were identified using GC–MS. The PubChem database (https://pubchem.ncbi.nlm.nih.gov/), accessed October 2023, was used to retrieve information such as cubic inch displacement (CID), formula, and structure data format (SDF) files. Energy minimization of ligands in *sdf* format was performed using OpenBabel software v2.3.1 for conversion into protein databank format (PDB) and for enhancing the flexibility of the atomic bonds that make up the ligand [23].

### Protein preparation

The candidate antimicrobial compounds inhibit the activity of bacteria and fungi, including *Bacillus subtilis*, *Escherichia coli*, *Staphylococcus aureus*, and *Candida albicans*. For in silico analysis, the protein targets for molecular docking were: filamenting temperature-sensitive mutant Z (FtsZ, PDB ID: 2VAM), a protein encoded by *ftsZ* that assembles into a ring at the site of future bacterial cell division (also called the Z ring) [24]; Aquaporin Z (PDB ID: 1RC2), a water channel protein in higher and lower organisms [25]; sortase A (SA) (PDB ID: 2MLM), a bacterial transpeptidase of bacterial cell wall proteins [26]; and acetohydroxyacid synthase (AHAS, PDB ID: 6DEK), an enzyme target for antimicrobial drug discovery that is considerably common in microbial synthesis pathways [27]. The 3D structure of the target was obtained from the RCSB PDB database (https://www.rcsb.org/), accessed in October 2023, in pdb format. Target optimization for the docking simulation was performed using the PyMOL software (v.2.5.2; Schrödinger, Inc., Washinton, USA) with an academic license for the removal of water molecules and native ligands. In this study, the targets superoxide dismutase 1 (SOD1) (PDB ID: 5YTU) and catalase (PDB ID: 1DGH) were used to bind candidate antioxidant compounds from *Cymbopogon* spp. Three-dimensional structures of each target were obtained from the Protein Data Bank (https://www.rcsb.org/), accessed in October 2023, *pdb* format [28].

### Drug-likeness prediction

This test is used to evaluate the characteristics of a query compound as a drug-like molecule by referring to several physicochemical rules using a specific method [29]. Lipinski’s Rule of Five is a drug-likeness prediction method based on molecular mass, high lipophilicity, hydrogen donor–acceptor interactions, and molar refractivity. In the present study, Lipinski's prediction was performed using the SCFBIO server (http://www.scfbio-iitd.res.cn/software/drugdesign/lipinski.jsp), accessed in October 2023, with a query compound *sdf* file [29].

### Virtual screening

Screening the activity of query compounds through the computational simulation of ligand-target binding is known as virtual screening [30]. In this study, we used molecular docking to identify the antimicrobial and antioxidant activities of *Cymbopogon* spp. by inhibiting FtsZ, Aquaporin Z, SA, and AHAS. Simulation was performed with the position of FtsZ autogrid docking from *Bacillus subtilis*: Center (Å) X: 28.973, Y: -5.776, Z: -2.052; Dimensions (Å) X: 67.136, Y: 55.679, Z: 64.620; Aquaporin Z from *Escherichia coli*: Center (Å) X: -33.395, Y: 33.776, Z: 10.766; Dimensions (Å) X: 44.594, Y: 34.944, Z: 53.370; SA from *Staphylococcus aureus*: Center (Å) X: 19.168, Y: 10.482, Z: 11.952; Dimensions (Å) X: 43.435, Y: 56.922, Z: 39.169; and AHAS from *Candida albicans* Center (Å) X: 64.379, Y: 247.139, Z: 45.709; Dimensions (Å) X: 63.263, Y: 53.140, Z: 63.241; SOD1 Center (Å) X: -73.660, Y: 25.713, Z: 18.286; Dimensions (Å) X: 69.194, Y: 75.585, Z: 41.691; and catalase Center (Å) X: 21.796, Y: 27.124, Z: 42.396; Dimensions (Å) X: 115.915, Y: 105.681, Z: 110.519 via PyRx software (v1.0.0; Scripps Research, USA) with an academic license. The structure of the ligand–protein complex was visualized using PyMOL (v.2.5.2; Schrödinger, Inc.) with an academic license for structural selection and single coloration [31].

### Ligand–protein analysis

Identification of the position and type of chemical bond interactions in ligand–protein molecular complexes was visualized through Discovery Studio Visualizer™ (v.16.1; Dassault Systèmes SE, France), accessed in October 2023. This software can visualize weak bond interactions, such as van der Waals, hydrogen, hydrophobic, pi/alkyl, and electrostatic interactions. Weak bonds in ligand–protein complexes trigger biological activities such as inhibitory responses. In this study, the positions of specific amino acid residues in the ligand-pocket binding domain were referred to as interaction hotspots [32].

### Molecular dynamic simulation

Chemical bond interaction stability at interaction hotspots was identified through molecular dynamic simulations using the CABS-flex server (v2.0; http://biocomp.chem.uw.edu.pl/CABSflex2/index), accessed in October 2023. The root-mean-square fluctuation (RMSF) is used to determine the stability of the molecular interactions. In this study, the RMSF refers to the conformational changes in amino acid residues at the interaction hotspot with a query ligand [33]. RMSF values were determined in CABS-flex (v2.0) based on rigidity, restraints, global c-alpha, side chain, number of cycles, cycles between trajectories, temperature range, and random number generator seed [34].

### Antimicrobial and antioxidant pathway prediction

The pathways of *Cymbopogon* spp.-derived compounds with more negative binding affinity values as antimicrobials and antioxidants were predicted using the SwissTargetPrediction server (http://www.swisstargetprediction.ch/), accessed October 2023, for target identification in *Homo sapiens*. Furthermore, pathway validation was performed through STRINGdb connected to Cytoscape software (v3.9.1). Cytoscape is used to identify interactions between query compounds and target proteins through molecular mechanisms [21].

### In vitro antioxidant activity

#### The 2,2-diphenyl-1-picryl-hydrazyl-hydrate (DPPH) inhibition assay

The DPPH inhibition assay was carried out as described previously [35]. Samples (100 μL) were diluted in methanol to concentrations of 1.075–200 µg/mL and mixed with 100 µL DPPH at 0.2 mM concentration. The mixture was then incubated at room temperature for 30 min. Ascorbic acid and trolox were used as positive controls. A microplate reader (Thermo Fisher Scientific, Waltham, MA, USA) was used to measure DPPH inhibition at 517 nm. The DPPH radical-scavenging capacity (%) was subsequently calculated using the following formula:

1 $$\frac{{A}_{control}-{A}_{sample}}{{A}_{control}}\times 100\%$$

where A_control_ represents the absorbance from the DPPH reagent and A_sample_ represents the absorbance of the DPPH reagent and sample mixture. The percentage of inhibition at each concentration was plotted and linearly regressed to obtain the half-maximal inhibitory concentration (IC_50_). The antioxidant activity of the plant extracts was determined based on IC_50_ value categorization: very strong (< 50 µg/mL), strong (50–100 µg/mL), moderate (101–250 µg/mL), weak (251–500 µg/mL), and no antioxidant activity (> 500 µg/mL) [36].

#### The 2,2’-azino-bis (3-ethylbenzothiazoline-6-sulphonic acid) (ABTS) inhibition assay

The ABTS assay was conducted as described previously [37]. ABTS (7 mM) and 2.4 mM potassium persulfate solutions were mixed to create the ABTS reagent. The mixture was left to sit for 12–16 h in the dark at room temperature. Absorbance of the reagent was measured at 734 nm. Next, 100 μL of samples were combined with 100 μL of the ABTS reagent at concentrations of 1.075–200 µg/mL. They were then incubated for 5 min at room temperature in the dark. The positive controls used were the same as those used for the DPPH assay: ascorbic acid and Trolox. After incubation, the absorbance was measured at 734 nm using a microplate reader (Thermo Fisher Scientific). The percent inhibition and IC_50_ values for the ABTS assay were calculated using the same formula as for the DPPH assay.

### In vitro cytotoxicity of *Cymbopogon* spp 

The cytotoxicity of all *Cymbopogon* spp. ethanolic extracts was evaluated using a modified version of the 3-[4, 5-dimethylthiazol-2-yl] 2, 5-diphenyl tetrazolium bromide (MTT) assay as described previously [21]. Hepatocyte-derived cellular carcinoma cells (Huh7it-1 cells) were grown at 37 °C in complete Dulbecco's modified Eagle's medium supplemented with 1% (v/v) glutamine (200 mM) at 5% CO_2_ and 95% humidity. The samples were dissolved in DMSO before dilution to different concentrations (0.1–1000 µg/mL). After 48-h incubation at 37 °C in 5% CO_2_ and 95% humidity, 5 mg/mL of MTT solution in phosphate buffered saline was added to each well. The cultures were then incubated for 4 h. Next, the solution was withdrawn, and 100 µL DMSO was added to dissolve the formazan crystals as MTT products. Cell viability was measured at 560 and 750 nm using a spectrophotometer. The half-cytotoxic concentration (CC_50_) was assessed by plotting the cell viability percentage and a series of concentrations to make the regression linear using Microsoft Excel (version 20.0; Microsoft Corporation, Redmond, Washington, USA). The experiment was performed in triplicate.

## Antimicrobial activity

### Antimicrobial assay: Preparation of media and inoculum

Four microbial strains, *B. substilis* TISTR 1248, *S. aureus* ATCC 25923*, E. coli* ATCC 25922, and *C. albicans* ATCC 10231, were tested using microbial assays. Solid and liquid media were prepared to conduct antimicrobial assays and maintain microbial cultures. Nutrient Agar (NA) and Potato Dextrose Agar (PDA) were used for the antibacterial and antifungal assays, respectively. In addition, NA and PDA were used to maintain the reference microbial culture in the reaction tubes. Nutrient Broth (NB) and isotonic NaCl solutions were used as liquid media. NB was used as the medium for bacterial strain subculture and preculture, whereas isotonic NaCl was only used for fungal strain preculture. The bacterial strains were precultured in culture bottles filled with sterile NB, incubated for 24 h at 37 °C, and diluted into 10% cultures before inoculation. The absorbance of 10% bacterial cultures was adjusted with sterile water using a spectrophotometer to reach the 0,5 McFarland standard. The fungal strain was precultured by homogenizing one loop of fungi from the reference culture into a culture bottle filled with sterile NaCl solution, and the culture was made immediately before inoculation [38].

### Well diffusion assay

The well diffusion assay was used to evaluate antimicrobial activity [39]. The antimicrobial activity of the extracts was determined based on the presence of inhibition zones on the surface of NA and PDA. The first 10 mL of agar medium was poured into sterilized Petri dishes and solidified as the first layer. Next, another 30 mL of agar was poured onto the solidified first layer and sat until it cooled but did not solidify. When the second layer cooled, 1 mL of bacterial culture was inoculated into NA, 1 mL of fungal culture was inoculated into PDA, and the second layer was placed again until it completely solidified. Four wells were then placed on the second solidified layer, into which the samples and controls were applied. The samples used were extracts diluted with 10% DMSO (250 and 500 mg/mL). DMSO (10%) and 1000 µg/mL chloramphenicol were used as the negative and positive controls, respectively, for the antibacterial assay. Similarly, nystatin (1000 µg/mL) was used as the positive control for the antifungal assay. The controls were tested on a separate agar media. Ten microliters of diluted samples were applied four times to each well. The plates were then incubated at 37 °C for 24 h for bacterial strains and 48 h for the fungal strain. After incubation, the plates were examined for the presence of inhibition zones on the agar surfaces. The diameter of the inhibition zone (DIZ, mm) was measured using Vernier calipers, and the mean was calculated and compared to that formed by the positive control. The percentage of inhibition (PI) was used to analyze the DIZ of the sample compared to the positive control and was calculated using the following formula (2):

2 $$PI \left(\%\right)=\frac{Mean zone of inhibition of the extract}{Zone of inhibition of the positive control}\times 100\%$$

### Statistical analyses

The data of antioxidant, antimicrobial, and cytotoxic activities are expressed as the mean ± standard deviation. The IC_50_ and CC_50_ values for in vitro antioxidant activity and cytotoxicity were calculated through linear regression using Microsoft Excel (version 20.0) [40]. Antioxidant, cytotoxicity, and antimicrobial data were analyzed using the Statistical Package for the Social Sciences (SPSS), version 18.0 (IBM Corp. SPSS Inc., Chicago, IL, USA).

## Result and discussion

### The extract yield and metabolite profile of* Cymbopogon* spp 

**Fig. 1:**
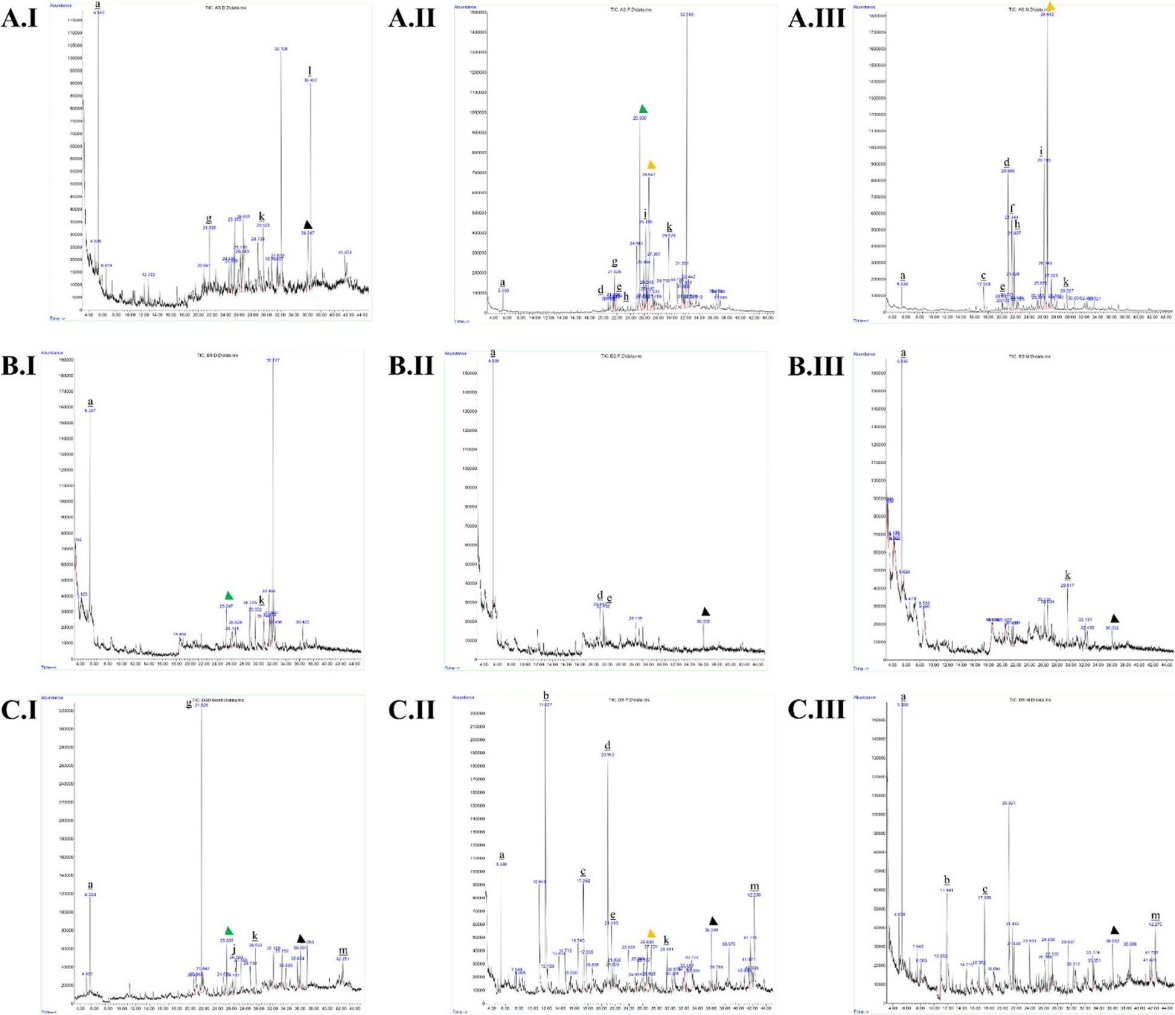
GC–MS chromatogram of *Cymbopogon spp*. ethanolic extract. **A** Roots, **B** stems, **C** leaves. I. Cymbopogon citratus, II. Cymbopogon nardus, III. Cymbopogon winterianus. a. Tetraethyl silicate; b. Geraniol; c. Methyleugenol; d. Benzene, 1,2-dimethoxy-4-(1-propenyl)-; e. Naphthalene, 1,2,3,4,4a,5,6,8a-octahydro-7-methyl-4-methylene-1-(1-methylethyl)-, (1.alpha.,4a.beta.,8a.alpha.)-; f. gamma.-Muurolene; g. Phenol, 2,5-bis(1,1-dimethylethyl); h. Naphthalene, 1,2,3,5,6,8a-hexahydro-4,7-dimethyl-1-(1-methylethyl)-, (1S-cis)-; i. tau.-Muurolol; j. (1S,4aS,7R,8aS)-1,4a-Dimethyl-7-(prop-1-en-2-yl)decahydronaphthalen-1-ol; k. 1-((1S,3aR,4R,7S,7aS)-4-Hydroxy-7-isopropyl-4-methyloctahydro-1H-inden-1-yl)ethanone; l. Benzenepropanoic acid, 3,5-bis(1,1-dimethylethyl)-4-hydroxy-, methyl ester; m. Phytol. Green arrow: Selin-6-en-4.alpha.-ol; yellow arrow: alpha.-Cadinol; black arrow: Hexadecanoic acid, methyl ester

The yields of crude ethanolic extracts of *Cymbopogon* spp. roots, stems, and leaves were approximately 11.40 ± 0.12 – 17.76 ± 0.2% (Additional file 1). GC–MS analysis revealed 53 metabolites from the extracts (Additional files 2 and Fig. 1); these were distributed evenly in every part of the *Cymbopogon* spp*.* organs. In contrast, 29, 13, and five metabolites were detected in only one, two, and three extracts, respectively. Additionally, only one metabolite was detected in four, five, six, seven, and nine extracts. 2,5-bis(1,1-dimethylethyl)- phenol (27.87%), alpha-cadinol (26.76%), and 1,2-dimethoxy-4-(1-propenyl)-benzene (20.56%) showed the highest percentage area; therefore, they were predicted to be major compounds. Previous studies have shown the bioactivity of some metabolites. Alpha-cadinol has antifungal, antimicrobial, and antioxidant activities [38, 39, 41, 42]. Similarly, selina-6-en-4-ol and n-hexadecanoic acid exhibit antimicrobial and antioxidant activities [43, 44]. Therefore, in silico and in vitro antimicrobial and antioxidant activities were determined for crude extracts of every *Cymbopogon* spp. organ. Computational pathway prediction of metabolites with antimicrobial activity is shown in Table 1.

**Table 1:** The selected metabolites of *Cymbopogon* spp. from GCMS as ligand information with PubChem database for computational analysis

No	RT (min)	Compound	CID	Formula	Area (%)
1	26.64	Alpha Cadinol	10,398,656	C_15_H_26_O	26.76
2	20.92	Benzene, 1,2-dimethoxy-4-(1-propenyl)-	6,425,292	C_12_H_16_O_3_	20.56
3	11.83	Citronellol	8842	C_10_H_20_O	17.15
4	36.46	Benzene, 1-(1,1-dimethylethyl)-4-methoxy-	94,750	C_11_H_16_O	13.50
5	25.30	Selina-6-en-4-ol	527,220	C_15_H_26_O	12.46
6	36.04	n-Hexadecanoic acid	985	C_16_H_32_O_2_	9.99
7	21.44	Gamma.Muurolene	6,432,308	C_15_H_24_	7.79
8	42.26	Phytol	5,280,435	C_20_H_40_O	6.46

*RT* retention time (minutes), *CID* cubic inch displacement

## In silico analysis of antioxidant and antimicrobial activity

### Ligand retrieval

Based on the GC–MS results of *Cymbopogon* spp., 8 compounds with the highest percent area > 3.26% were used as ligands. Information on the CID and formula of each compound was obtained from PubChem (Table 1), for drug-likeness prediction analysis before conducting in silico analysis of the antioxidant and antimicrobial activity assays.

### Drug-likeness analysis

Drug likeness assesses the physicochemical properties of query compounds containing drug molecules. Several parameters, including molecular mass, high lipophilicity, hydrogen bond donors, hydrogen bond acceptors, and molar refractivity, were used for drug-like molecule determination in this study. Query compounds with positive predictions as drug-like molecules must fulfill at least two Lipinski rules. In the present study, all compounds from *Cymbopogon* spp. were drug-like molecules (Table 2), as predicted based on Lupinski’s Rule of Five.

**Table 2:** The results of drug-likeness prediction

No	Compounds	MM (≤ 500 D)	LogP (≤ 5)	HBD (≤ 5)	HBA (≤ 10)	MR (40 -130)	Probable
1	Alpha Cadinol	222.000	3.775	1	1	68.156	Drug-like molecule
2	Benzene, 1,2-dimethoxy-4-(1-propenyl)-	208.000	2.711	0	3	60.205	Drug-like molecule
3	Citronellol	156.000	2.751	1	1	49.531	Drug-like molecule
4	Benzene, 1-(1,1-dimethylethyl)-4-methoxy-	164.000	2.992	0	1	51.693	Drug-like molecule
5	Selina-6-en-4-ol	222.000	3.919	1	1	68.226	Drug-like molecule
6	n-Hexadecanoic acid	256.000	5.552	1	2	77.947	Drug-like molecule
7	Gamma Muurolene	204.000	4.581	0	0	66.672	Drug-like molecule
8	Phytol	296.000	6.364	1	1	95.561	Drug-like molecule

*MM* molecular mass, *LogP* high lipophilicity, *HBD* hydrogen bond donors, *HBA* hydrogen bond acceptors, *MR* molar refractivity

### Virtual analysis

**Fig. 2:**
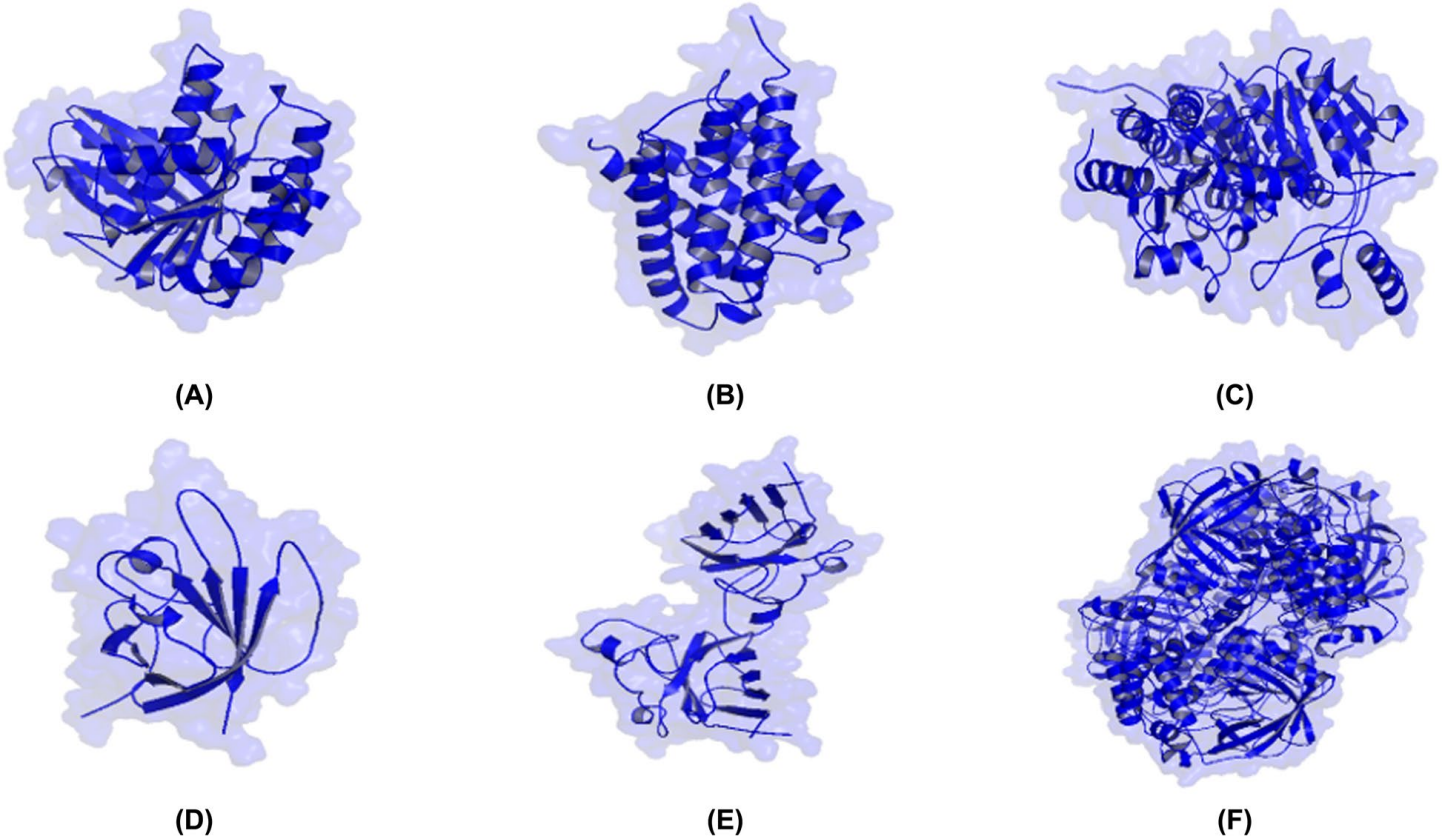
Three-dimensional structure visualization of target proteins. **A** *Bacillus subtilis*-FtsZ, **B** *Escherichia coli*-Aquaporin Z, **C** *Candida albicans*-AHAS, **D** *Staphylococcus aureus*-SA, **E** SOD1, and **F** Catalase

Virtual analysis or molecular docking is used to identify bonding interaction patterns and ligand activity on a target. Ligand activity is indicated by the binding affinity value of the ligand–protein complex [28]. Moreover, antioxidant and antimicrobial activities are related to pathway mechanisms regulated by proteins, such as the filamenting mutant Z (FtsZ) protein, the MciZ synthetic peptide [45], aquaporin Z [46], SA [47], acetohydroxy acid or AHAS [48], SOD1, and catalase [49]. The structures of FtsZ, aquaporin Z, AHAS, SA, SOD1, and catalase are displayed as transparent surfaces, cartoons, and single colors in Fig. 2.

**Fig. 3:**
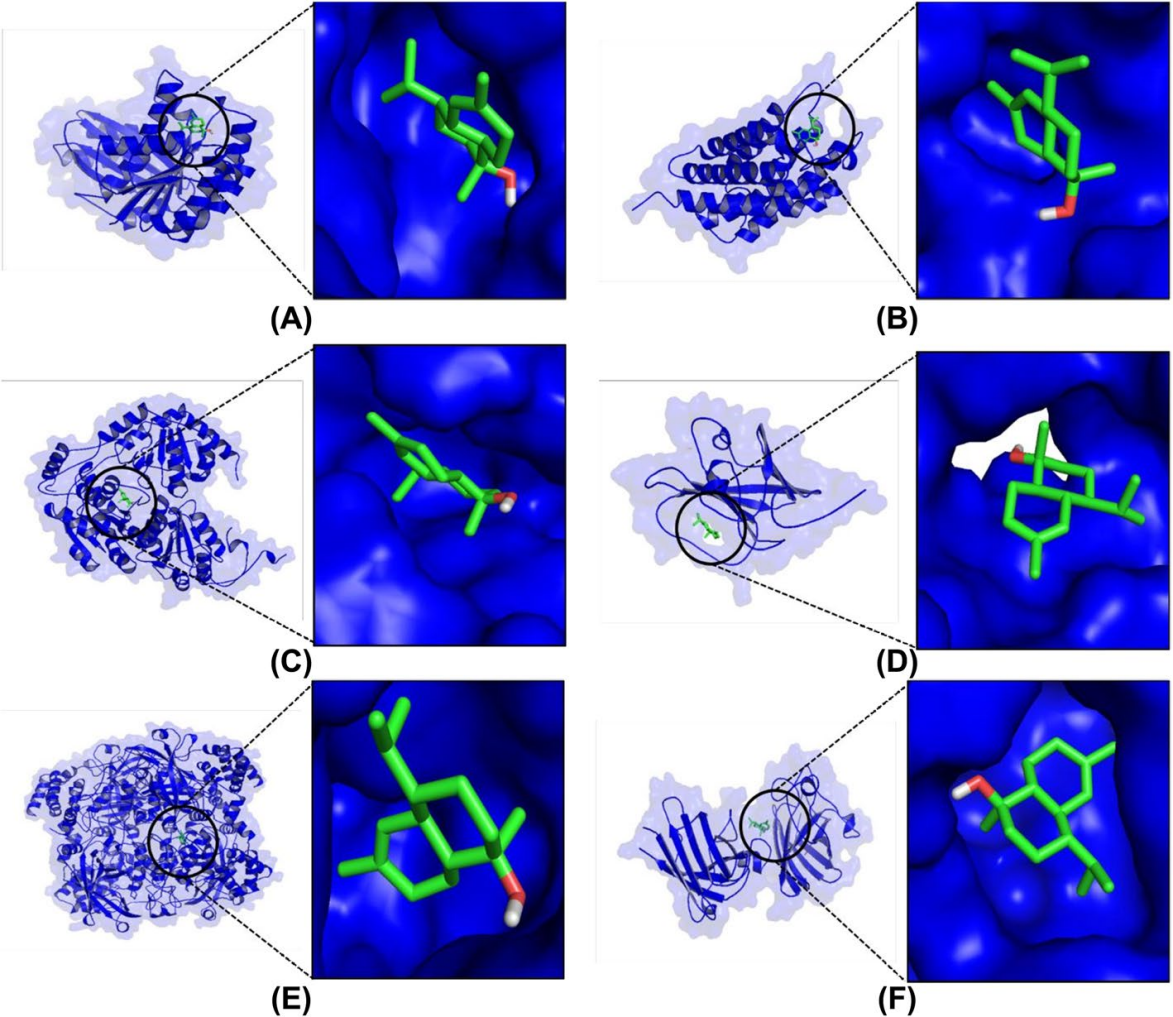
Ligand–protein visualization from molecular docking simulation. **A** Alpha Cadinol-FtsZ, **B** Alpha Cadinol-Aquaporin Z, **C** Alpha Cadinol-AHAS, **D** Alpha Cadinol-SA, **E** Alpha Cadinol-SOD1, and **F** Alpha Cadinol-catalase

Binding affinity is the negative binding energy formed when interactions between molecules refer to the thermodynamic rule. When the value of binding affinity is more negative, the ligand activity increases; this makes it possible to trigger biological responses such as inhibition [50]. In the present study, molecular docking was used to screen for the antibacterial activity of *Cymbopogon* spp. through the binding of FtsZ, Aquaporin Z, AHAS, SA, SOD, and catalase. The results of docking simulation showed that alpha-cadinol has the most negative binding affinity to FtsZ, aquaporin Z, AHAS, SA, SOD1, and catalase (Table 3). This metabolite is predicted to act as an antibacterial agent by inhibiting three targets, namely FtsZ, aquaporin Z, and SA from *B. subtilis*, *E. coli*, and *S. aureus*. Moreover, it acts as an antifungal agent by inhibiting AHAS in *C. albicans*. The inhibition of SOD and catalase by alpha-cadinol showed better antioxidant activity than other compounds from *Cymbopogon* spp. Figure 3 shows the three-dimensional structures of the following ligand–protein complexes: alpha cadinol-FtsZ, alpha cadinol-aquaporin Z, alpha cadinol-AHAS, alpha cadinol-SA, alpha cadinol-SOD1, and alpha cadinol-catalase.

**Table 3:** Binding affinity of *Cymbopogon* spp. compounds

No	Compound	Binding Affinity (kcal/mol)					
		***Bacillus subtilis* (FtsZ)**	***Escherichia coli* (Aquaporin Z)**	***Candida albicans* (AHAS)**	***Staphylococcus aureus* (SA)**	**SOD**	**Catalase**
1	Alpha Cadinol	**-6.4**	**-6.0**	**-7.0**	**-6.5**	**-6.5**	**-7.9**
2	Benzene, 1,2-dimethoxy-4-(1-propenyl)-	-5.6	-5.5	-6.4	-5.4	-5.1	-6.8
3	Citronellol	-4.7	-4.9	-5.4	-4.9	-4.8	-6.3
4	Selina-6-en-4-ol	-6.0	-5.4	-6.9	-6.3	-6.0	-7.5
5	Benzene, 1-(1,1-dimethylethyl)-4-methoxy-	-5.7	-5.4	-6.3	-5.3	-5.2	-6.6
6	n-Hexadecanoic acid	-4.6	-5.4	-4.6	-4.9	-5.0	-4.8
7	Gamma Muurolene	-6.1	-5.9	-6.7	-6.0	-6.3	-7.1
8	Phytol	-4.7	-6.0	-5.9	-5.7	-5.1	-5.6

**Fig. 4:**
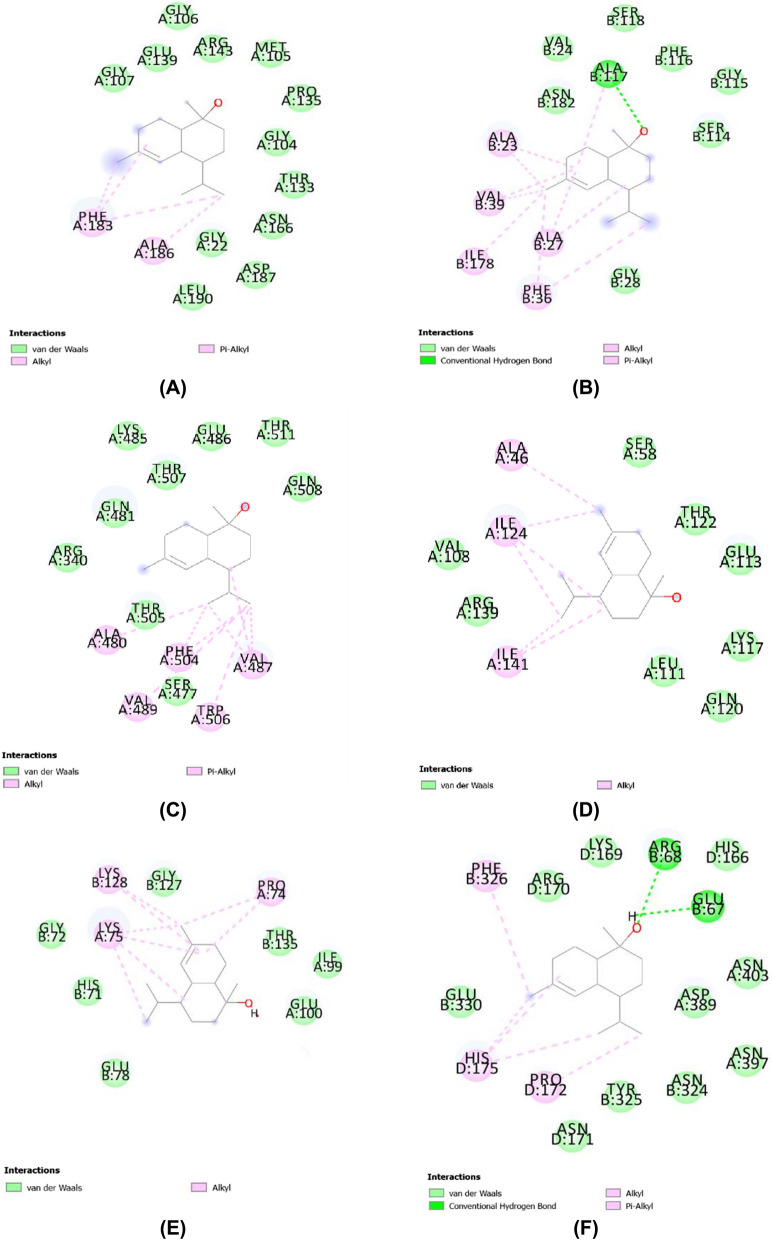
Two-dimensional visualization of ligand–protein interactions. **A** Alpha Cadinol-FtsZ, **B** Alpha Cadinol-Aquaporin Z, **C** Alpha Cadinol-AHAS, **D** Alpha Cadinol-SA, **E** Alpha Cadinol-SOD1, and **F** Alpha Cadinol-catalase

Weak bond interactions trigger target-specific biological activities such as inhibition. For example, van der Waals, hydrogen, hydrophobic, pi/alkyl, and electrostatic bonds can play a role in the inhibitory activity of the target [34]. In this study, alpha-cadinol interacted with FtsZ via van der Waals interactions at Gly107, Glu139, Gly106, Arg143, Met105, Pro135, Gly104, Thr133, Asn166, Gly22, Asp187, and Leu190 and via alkyl/pi interactions at Phe183 and Ala186. Moreover, alpha-cadinol interacted with Aquaporin Z via van der Waals interactions at Asn182, Val24, Ser118, Phe116, Gly115, Ser114, and Gly28; hydrogen interactions at Ala117; and alkyl/pi interactions at Ala117, Ala23, Val39, Ala27, Ile178, and Phe36. This metabolite further interacted with AHAS via van der Waals interactions at Arg340, Gln481, Thr507, Lys485, Glu486, Thr511, Gln508, Thr505, and Ser477 and via alkyl/pi interactions at Ala480, Phe504, Val489, Trp506, and Val487. Additionally, alpha-cadinol interacted with SA via van der Waals interactions at Ser58, Thr122, Glu113, Lys117, Leu111, Gln120, Val108, and Arg139, and via alkyl/pi interactions at Ala46, Ile124, and Ile141. Alpha-cadinol interacted with SOD1 via van der Waals interactions at Glu78, His71, Gly72, Gly127, Thr135, Ile99, and Glu100 and via alkyl/pi interactions at Lys128, Lys75, and Pro74. Finally, alpha-cadinol interacted with catalase via van der Waals interactions at Glu330, Asn171, Tyr325, Asn324, Asn397, Asp389, Asn403, His166, Lys169, and Arg170, and via alkyl/pi/sigma interactions at Phe326, His175, and Pro172 (Fig. 4).

**Fig. 5:**
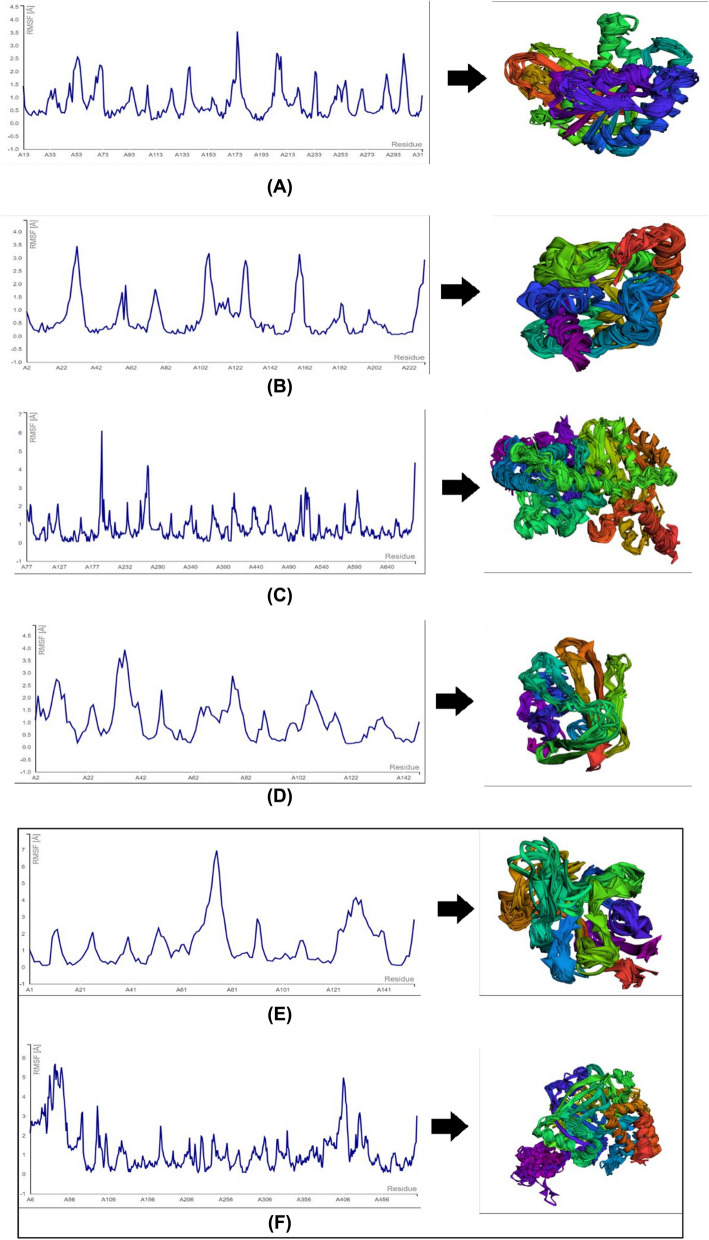
Root Mean Square Fluctuation (RMSF) dynamic plot and conformational structures of target proteins. **A** Alpha Cadinol-FtsZ, **B** Alpha Cadinol-Aquaporin Z, **C** Alpha Cadinol-AHAS, **D** Alpha Cadinol-SA, **E** Alpha Cadinol-SOD1, and **F** Alpha Cadinol-catalase

The RMSF values at the ligand–protein complex hotspots in this study consisted of van der Waals forces (1.485, 2.188, 0.797, 0.627, 0.432, 0.578, 0.489, 0.133, 0.372, 0.392, 0.194, and 0.128) and alkyl/pi interactions (0.183 and 0.186) in the FtsZ domain (MD plot link: https://biocomp.chem.uw.edu.pl/CABSflex2/job/4cbe0f320ffa1fa/), accessed April 2024. RMSF on the Aquaporin Z domain (MD plot link: https://biocomp.chem.uw.edu.pl/CABSflex2/job/c7edfbf8a9bc31/), accessed April 2024, occurred via van der Waals forces (0.725, 0.707, 1.478, 1.020, 1.276, 1.110, and 0.328), hydrogen bonds (1.196), and alkyl/pi interactions (1.196, 0.523, 0.142, 1.442, 0.514, and 0.365). RMSF in the AHAS domain (MD plot link: https://biocomp.chem.uw.edu.pl/CABSflex2/job/9d455305023de11/), accessed April 2024, occurred via van der Waals (1.471, 0.641, 1.326, 1.021, 0.715, 0.678, 1.971, 0.907, and 0.400) and alkyl/pi (0.480, 0.687, 0.489, 1.025, and 0.4871) interactions. The RMSF in the SA domain (MD plot link: https://biocomp.chem.uw.edu.pl/CABSflex2/job/ea22bce9fb5421d/), accessed April 2024, occurred via van der Waals (0.310, 0.153, 0.709, 1.161, 1.221, 0.175, 2.011, and 0.351) and alkyl/pi (0.356, 0.193, and 0.337) interactions. RMSF in the SOD1 domain (MD plot link: https://biocomp.chem.uw.edu.pl/ CABSflex2/job/5eadcf318a44b3/), accessed April 2024, occurred via van der Waals (2.863, 2.596, 1.440, 0.239, 2.439, 0.509, and 0.485) and alkyl/pi (2.997, 0.540, and 2.156) interactions. Finally, RMSF in the catalase domain (MD plot link: https://biocomp.chem.uw.edu.pl/CABSflex2/job/d89e8a25c3981f4/), accessed April 2024, occurred through van der Waals (0.829, 0.914, 1.137, 1.277, 1.452, 1.686, 2.990, 0.686, 0.703, and 0.768) and alkyl/pi (1.161, 1.310, and 1.639) interactions (Fig. 5).

The stability of molecular interactions at the hotspots was identified using molecular dynamics simulations. The stability of bonding interactions at hotspots is indicated by RMSF values < 3 (Å). RMSF refers to the deviation in the interaction distance formed by the ligand in the target domain [30]. The alpha cadinol interactions of *Cymbopogon* spp*.* formed stable interactions on FtsZ, Aquaporin Z, SA, AHAS, SOD1, and catalase domains with RMSF values < 3 (Å). This indicates that the compound has antibacterial, antifungal, and antioxidant properties.

**Fig. 6:**
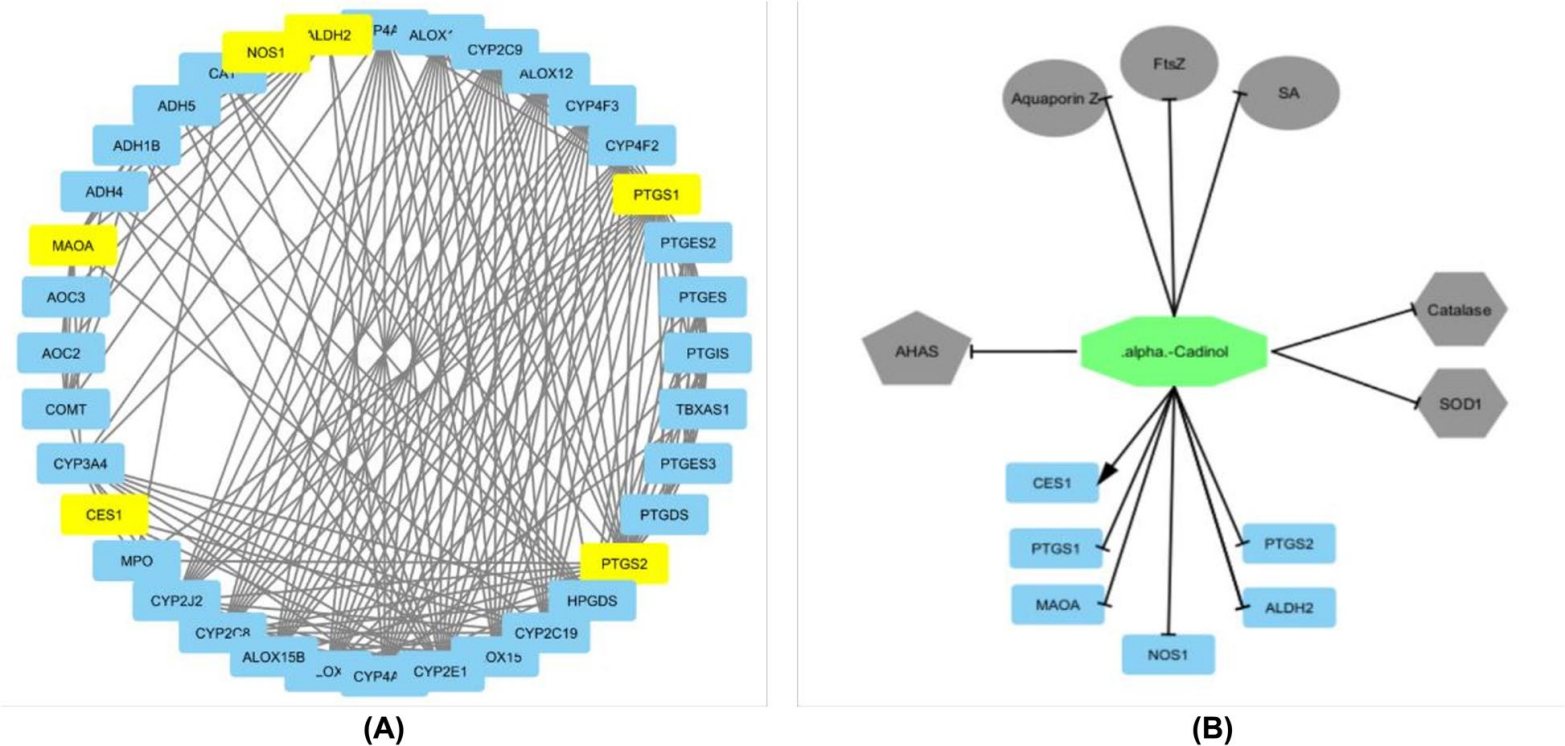
Prediction of antioxidant and antimicrobial activity pathways of alpha cadinol. **A** Target position of alpha cadinol from the pathway database. **B** Antibacterial, antifungal, and antioxidant pathway of alpha cadinol compounds. The T shape on the target indicates inhibitory activity and arrows for activation. The yellow color of the nodes indicates the target position. FtsZ: Filamenting temperature-sensitive mutant Z; SA: Sortase A; AHAS: Acetohydroxyacid synthase; SOD: Superoxide dismutase 1; PTGS2: Cyclooxygenase-2; PTGES: Prostaglandin E synthase; PTGIS: Prostaglandin I2 synthase; TBXAS1: Thromboxane A synthase 1; PTGES3: Prostaglandin E synthase 3; PTGDS: Prostaglandin D2 synthase; HPGDS: Hematopoietic prostaglandin D synthase; CYP2C19: Cytochrome P450 family 2 subfamily C member 19; CYP2E1: Cytochrome P450 family 2 subfamily E member 1; ALOX15B: Arachidonate 15-lipoxygenase type B; CYP2C8: Cytochrome P450 family 2 subfamily C member 8; CYP2J2: Cytochrome P450 family 2 subfamily J member 2; MPO: Myeloperoxidase; CYP2C9: Cytochrome P450 family 2 subfamily C member 9; ALOX12: Arachidonate 12-lipoxygenase; CYP4F3: Cytochrome P450 family 4 subfamily F member 3; CYP4F2: Cytochrome P450 family 4 subfamily F member 2; PTGS1: Cyclooxygenase-1; NOS1: Nitric-oxide synthase; ADH4: Alcohol dehydrogenase 4; ADH1B: Alcohol dehydrogenase 1B; ADH5: Alcohol dehydrogenase 5; CAT: Catalase; MAOA: Monoamine oxidase B; AOC3: Amine oxidase copper containing 3; AOC2: Amine oxidase copper containing 2; COMT: Catechol-O-methyltransferase; CYP3A4: Cytochrome P450 family 3 subfamily A member 4; ALDH2: Aldehyde dehydrogenase; CES1: Carboxylesterase 1

Target prediction showed that alpha cadinol from *C. citratus* has other targets for antioxidant activity, namely cyclooxygenase-2 (PTGS2), cyclooxygenase-1 (PTGS1), nitric-oxide synthase (NOS1), monoamine oxidase B (MAOA), aldehyde dehydrogenase (ALDH2), and carboxylesterase 1 (CES1). Alpha-cadinol exhibited inhibitory activity against PTGS1, PTGS2, NOS1, MAOA, and ALDH2. CES1 can also be activated by alpha-cadinol as an antioxidant pathway, additionally annotating the pathway with FtsZ, Aquaporin Z, SA, and AHAS inhibitory targets for antibacterial and antifungal activity (Fig. 6A). PTGS1, PTGS2, NOS1, MAOA, and ALDH2 increase ROS production [51–55]. In contrast, CES1 reduces ROS production under oxidative stress [56]. The target proteins of alpha-cadinol include PTGS1, PTGS2, NOS1, MAOA, and ALDH2. In the biological pathways of *Homo sapiens*, nodes with hexagonal, pentagonal, and elliptical shapes were the targets obtained during docking analysis. Additional pathway prediction targets from the database are shown as nodes with rounded rectangular shapes (Fig. 6B). The pathway consisting of antibacterial, antifungal, and antioxidant drugs has a confidence value of 0.6 (medium confidence) or 70–80%, which is highly accurate [54–56].

### In vitro antioxidant activity

DPPH and ABTS assays were performed to assess the antioxidant activities of the extracts. The IC_50_ values of the crude extracts prepared using each solvent are listed in Table 4. The leaves of *Cymbopogon* spp*.* had strong antioxidant activity, which was determined based on the Prieto criteria [28]. IC_50_ values of ABTS were 40.90 ± 0.94, 44.86 ± 1.23, 47.04 ± 1.03, 75.93 ± 1.48, 77.44 ± 1.20, 80.53 ± 1.38, 85.46 ± 21.17, 103.45 ± 6.69, and 128.93 ± 18.49 µg/mL for *C. nardus* leaves, *C. citratus* leaves, *C. winterianus* leaves, *C. citratus* stems, *C. winterianus* stems, *C. winterianus* roots, *C. nardus* roots, *C. nardus* stems, and *C. citratus* roots, respectively. Contrastingly, the IC_50_ values of DPPH were 61.30 ± 1.04, 86.36 ± 1.09, 92.87 ± 1.54, 101 ± 13.01, 131.54 ± 10.74, 152.46 ± 10.79, 162.97 ± 18.83, 309.74 ± 6.48, and 346.19 ± 13.36 µg/mL for *C. winterianus* leaves, *C. citratus* leaves, *C. nardus* leaves, *C. winterianus* stems*, C. nardus* stems*, C. citratus* stems, *C. winterianus* roots, *C. nardus* roots, and *C. citratus* roots, respectively. The IC_50_ of ABTS was lower than that of DPPH. Overall, the ABTS and DPPH IC_50_ values of the leaf extract (strong) were the lowest, followed by those of the stem (moderate) and root (moderate-weak) extracts [28]. The potent antioxidant activity of the ethanolic extract of *Cymbopogon* spp. was likely due to the presence of bioactive compounds (Table 1, Additional File 2). Compared to other studies that have previously reported high antioxidant compounds, the antioxidant activity of ethanolic extracts from *Cymbopogon* spp. was higher than that of *Sonchus arvensis* L. leave [30] and *Pterocarpus macrocarpus* Kurz. bark extracts [29].

**Table 4:** Half-maximum inhibition concentration (IC_50_) of three commercial *Cymbopogon* spp. Extracts

Sample	Part of plant	IC_50_ (µg/mL)	
		DPPH*	ABTS*
*Cymbopogon winterianus*	Leaves	61.30 ± 1.04^a^	47.04 ± 1.03^b^
	Stems	101 ± 13.01^ab^	77.44 ± 1.20^c^
	Roots	162.97 ± 18.83^de^	80.53 ± 1.38^cd^
*Cymbopogon nardus*	Leaves	92.87 ± 1.54^ab^	40.90 ± 0.94^a^
	Stems	131.54 ± 10.74^cd^	103.45 ± 6.69^cd^
	Roots	309.74 ± 6.48^e^	85.46 ± 21.17^cd^
*Cymbopogon citratus*	Leaves	86.36 ± 1.09^b^	44.86 ± 1.23^b^
	Stems	152.46 ± 10.79^de^	75.93 ± 1.48^c^
	Roots	346.19 ± 13.36^f^	128.93 ± 18.49^d^
Positive Control	Trolox	0.86 ± 0.97	0.97 ± 0.30
	Ascorbic acid	2.77 ± 1.30	5.12 ± 2.43
P value		0.001	0.002

All data is represented as the mean ± standard deviation (*SD*) of three independent experiments; *different superscript letters (^a−f^) indicate a significant difference between each sample in a group of DPPH or ABTS (Nonparametric test using Kruskal–Wallis, significant level is 0.05)

The IC_50_ values for *C. citratus.* leaf ethanolic extract in the present study were lower against ABTS than those previously reported for *C. citratus* leaf methanolic extract and fractions using *n*-hexane, chloroform, and ethyl acetate [57]. This indicates the higher antioxidant activity of *C. citratus* extracts in the present study. Compared to previously reported *C. nardus* essential oil [58], *C. nardus* leaf ethanolic extracts showed higher IC_50_ values against DPPH in the present study. Moreover, the IC_50_ values for all *C. winterianus* leaf ethanolic extracts against ABTS and DPPH were lower than those of the essential oil extracted from *C. winterianus* leaves [59]. In contrast, the IC_50_ values of *Cymbopogon* spp. root and stem ethanolic extracts were higher than those previously reported for *Cymbopogon* spp. leaf extracts [57–59].

## In vitro cytotoxicity of *Cymbopogon* spp ethanolic extracts

The toxicity of *Cymbopogon* spp. ethanolic extracts to Huh7it-1 cells was assessed using an MTT assay (Table 5). *C. citratus* leaf extract had the lowest cytotoxic effect on the human hepatocyte cells (1421.07 ± 20.88 µg/mL), followed by *C. citratus* root (822.18 ± 36.45 µg/mL), *C. winterianus* leaf (768.79 ± 74.19 µg/mL), *C. nardus* root (753.81 ± 40.44 µg/mL), *C. winterianus* root (621.81 ± 10.59 µg/mL), *C. winterianus* stem (555.71 ± 44.72 µg/mL), *C. citratus* stem (477.75 ± 4.01 µg/mL), *C. nardus* leaf (437.39 ± 7.47 µg/mL), and *C. nardus* stem (426.74 ± 26.05 µg/mL) extracts. The 50 percent cytotoxic concentration (CC_50_) is defined as the concentration of the sample compound required to reduce cell viability by 50% [60]. United States National Cancer Institute (US NCI) plant screening program, crude extract is considered cytotoxic when CC_50_ is lower than 30 µg/mL. This study showed that the CC_50_ of all extracts were higher than 30 µg/mL, which means that all extracts were low toxicity [61].

**Table 5:** The toxicity result of three commercial *Cymbopogon* spp. extracts

Sample	Part of plant	CC_50_ (Concentration µg/mL)*
*Cymbopogon winterianus*	Leaves	768.79 ± 74.19^cd^
	Stems	555.71 ± 44.72^b^
	Roots	621.81 ± 10.59^c^
*Cymbopogon nardus*	Leaves	437.39 ± 7.47^a^
	Stems	426.74 ± 26.05^a^
	Roots	753.81 ± 40.44^cd^
*Cymbopogon citratus*	Leaves	1421.07 ± 20.88^e^
	Stems	477.75 ± 4.01^a^
	Roots	822.18 ± 36.45^d^
*P* value		0.001

All data is represented as the mean ± standard deviation (*SD*) of three independent experiments; *different superscript letters (^a−d^) indicate a significant difference between each sample in a group (Nonparametric test using Kruskal–Wallis, significant level is 0.05)

## Antimicrobial activity

Antimicrobial tests against bacteria and yeasts were performed using all crude extracts. All *C. citratus* extracts, except the root extract at 250 mg/mL and the leaf extract at both concentrations, showed active antibacterial activity against *B. subtilis* (Table 6), with a PI of 19.58 ± 1.72%, 17.27 ± 0.77%, and 17.75 ± 0.48% for the root extract at 500 mg/mL and the stem extract at 250 and 500 mg/mL, respectively. All *C. nardus* extracts exhibited antibacterial activity against *B. subtilis* (Table 6) (50.57 ± 2.92% and 52.33 ± 6.91% inhibition by 250 and 500 mg/mL root extracts, 34.99 ± 3.00% and 45.93 ± 7.62% by 250 and 500 mg/mL stem extracts, and 39.43 ± 4.37% and 51.93 ± 2.19% by 25% and 50% leaf extracts, respectively). Similarly, *C. winterianus* extracts showed antibacterial activity against *B. subtilis* (Table 6) (22.45 ± 1.28% and 28.28 ± 2.59% inhibition at 250 and 500 mg/mL root extracts, 48.09 ± 1.42% and 64.35 ± 1.50% at 25% and 50% stem extracts, and 19.97 ± 3.41% and 27.69 ± 3.94% at 250 and 500 mg/mL leaf extracts, respectively). The *C. nardus* root (89.38 ± 2.42% and 96.41 ± 2.61% at 250 and 500 mg/mL), stem (28.98 ± 0.30% and 32.03 ± 1.08% at 250 and 500 mg/mL), and leaf (76.70 ± 3.55% and 86.72 ± 3.15% at 250 and 500 mg/mL) extracts showed excellent antibacterial activity against *S. aureus*. Additionally, the stem extracts of *C. citratus* (28.98 ± 0.30% and 32.03 ± 1.08% at 250 and 500 mg/mL) and *C. winterianus* (48.08 ± 5.74% and 64.46 ± 5.40% at 250 and 500 µg/mL) showed antibacterial activity. Only *C. nardus* extracts showed active antibacterial activity against *E. coli*, with a PI of 88.97 ± 5.71% and 127.24 ± 5.03% at 25% and 50% root extracts, 47.10 ± 4.01% and 68.01 ± 5.84% at 250 and 500 mg/mL stem extracts, and 105.56 ± 7.41% and 124.85 ± 6.09% at 250 and 500 mg/mL leaf extracts. Of all extracts tested against *C. albicans*, only the root extracts of *C. nardus* showed active antifungal activity, with a PI of 23.61 ± 1.26% and 24.33 ± 1.23% at 250 and 500 mg/mL, respectively.

**Table 6:** Diameter of inhibition zone (DIZ) and percentage of inhibition (PI) of *C. citratus* ethanolic extract against *Bacillus subtilis*, *Staphylococcus aureus*, *Escherichia coli*, and *Candida albicans*

Bacteria	Part of Plant	Concentration (mg/mL)	*Cymbopogon citratus*		*Cymbopogon winterianus*		*Cymbopogon nardus*	
			**Diameter of inhibition zone (DIZ) (mm)**	**Percentage of inhibition (PI) (%)**	**Diameter of inhibition zone (mm)**	**Percentage of inhibition (%)**	**Diameter of inhibition zone (DIZ), (mm)**	**Percentage of inhibition (PI) (%)**
*Bacillus substilis*	Root	250	ND	ND	8.95 ± 0.51^f^	22.45 ± 1.28^e^	20.17 ± 1.16^b^	50.57 ± 2.92^a^
		500	7.8 ± 0.68^b^	19.58 ± 1.72^a^	11.28 ± 1.03^d^	28.28 ± 2.59^c^	20.87 ± 2.76^b^	52.33 ± 6.91^a^
	Stem	250	6.89 ± 0.31^c^	17.27 ± 0.77^b^	18.38 ± 0.56^c^	48.09 ± 1.42^b^	13.96 ± 1.19^c^	34.99 ± 3.00^c^
		500	7.08 ± 0.68^c^	17.75 ± 0.48^b^	25.00 ± 0.60^b^	64.35 ± 1.50^a^	18.32 ± 3.04^bc^	45.93 ± 7.62^ab^
	Leaves	250	ND	ND	7.96 ± 1.36^ef^	19.97 ± 3.41^de^	15.73 ± 1.74^ cd^	39.43 ± 4.37^bc^
		500	ND	ND	11.04 ± 1.57^de^	27.69 ± 3.94^ cd^	20.71 ± 0.87^b^	51.92 ± 2.19^a^
	Chloramphenicol	1000	39.88 ± 0.32^a^	-	39.88 ± 0.32^a^	-	39.88 ± 0.32^a^	-
*Staphylococcus aureus*	Root	250	ND	ND	ND	ND	22.90 ± 0.62^b^	89.38 ± 2.42^b^
		500	ND	ND	ND	ND	24.70 ± 0.67^a^	96.41 ± 2.61^a^
	Stem	250	7.45 ± 0.08^c^	28.98 ± 0.30^b^	12.32 ± 1.47^c^	48.08 ± 5.74^b^	17.68 ± 2.09^c^	68.99 ± 8.17^c^
		500	8.21 ± 0.28^b^	32.03 ± 1.08^a^	16.52 ± 1.38^b^	64.46 ± 5.40^a^	19.30 ± 1.84^c^	75.33 ± 7.18^c^
	Leaves	250	ND	ND	ND	ND	19.65 ± 0.91^c^	76.70 ± 3.55^c^
		500	ND	ND	ND	ND	22.22 ± 0.81^b^	86.72 ± 3.15^b^
	Chloramphenicol	1000	25.62 ± 0.28^a^	-	25.62 ± 0.28^a^	-	25.62 ± 0.28^a^	-
*Escherichia coli*	Root	250	ND	ND	ND	ND	23.16 ± 3.90^c^	88.97 ± 5.71^c^
		500	ND	ND	ND	ND	31.34 ± 1.24^a^	127.24 ± 5.03^a^
	Stem	250	ND	ND	ND	ND	11.60 ± 0.98^e^	47.10 ± 4.01^e^
		500	ND	ND	ND	ND	15.03 ± 1.44^d^	68.01 ± 5.84^d^
	Leaves	250	ND	ND	ND	ND	26.00 ± 1.83^b^	105.56 ± 7.41^b^
		500	ND	ND	ND	ND	30.75 ± 1.50^a^	124.85 ± 6.09^a^
	Chloramphenicol	1000	24.63 ± 0.80	-	24.63 ± 0.80	-	24.63 ± 0.80^bc^	-
*Candida albicans*	Root	250	ND	ND	ND	ND	8.62 ± 0.46^b^	23.61 ± 1.26^a^
		500	ND	ND	ND	ND	8.88 ± 0.45^b^	24.33 ± 1.23^a^
	Stem	250	ND	ND	ND	ND	ND	ND
		500	ND	ND	ND	ND	ND	ND
	Leaves	250	ND	ND	ND	ND	ND	ND
		500	ND	ND	ND	ND	ND	ND
	Nystatin	1000	36.50 ± 0.07	-	36.50 ± 0.07	-	36.50 ± 0.07^a^	-

All data is represented as the mean ± standard deviation (*SD*) of three independent experiments; *different superscript letters (^a−c^) indicate a significant difference between each sample in a group of diameter of inhibition zone (*DIZ*) or percentage of inhibition (*PI*) (Nonparametric test using Kruskal–Wallis, significant level is 0.05). *ND*: Not detected

*C. citratus* extracts showed no significant inhibition zones against *E. coli* and *C. albicans*. In contrast, they had a considerable effect against *B. subtilis* and *S. aureus*. In a previous study, *C. citratus* essential oil exhibited stronger antibacterial activity against gram-positive bacteria (*B. subtilis* and *S. aureus*) than against gram-negative bacteria (*E. coli*) [59]. Additionally, *S. aureus* was less resistant to antimicrobials than *B. subtilis* in the present study, which is consistent with previous findings [9]. The contrast in activity between *C. citratus* against *E. coli* and *S. aureus* is also comparable to a similar test carried out by Subramaniam et al., in which *C. citratus* root and leaf extracts exhibited stronger antimicrobial activity against *S. aureus* than against *E. coli* [9]. However, only the stems exhibited antibacterial activity against *S. aureus* in the present study, and there was no activity against *E. coli*. Moreover, antifungal activity against *C. albicans* was insignificant. This is contrary to the findings of Pratama and Permana [13], who reported that *C. citratus* essential oils showed significant inhibition zones at 200, 300, and 500 mg/mL.

All *C. nardus* extracts showed substantial inhibition zones against *B. subtilis*, *S. aureus*, and *E. coli*, whereas only the root extract showed inhibition zones against *C. albicans*. The root extracts exhibited higher antibacterial activity against gram-positive bacteria, followed by the leaf and stem extracts. In contrast, the leaf extracts were more effective against gram-negative bacteria, followed by the root and stem extracts. The antimicrobial activity of *C. nardus* extracts is related to the presence of terpenoids (especially monoterpenes) and phenolic compounds. Kamal et al. [13] reported that *C. nardu*s essential oil has antibacterial activity against both gram-positive and gram-negative bacteria because of the presence of citronellal and z-citral, two of the 13 monoterpenes identified in the essential oil. In the present study, all *C. nardus* extracts exhibited strong antibacterial activity against both gram-positive and gram-negative bacteria. Antibacterial activity was comparable to that reported by Yunita et al [62], in which the leaf extract of *C. nardus* showed a high response against *S. aureus* at 20 mg/mL. Similarly, Shintawati et al. [63] showed that *S. aureus* is less susceptible to the *C. nardus* oil obtained from leaves than *E. coli*, which was also reflected in our study. Antifungal activity was due to the presence of citronellal [13]. However, *C. nardus* root extract exhibited weaker antifungal activity in the present study, as demonstrated by the smaller inhibition zone observed against *C. albicans*.

*C. winterianus* extracts showed no significant antimicrobial activity against *E. coli* and *C. albicans*. Rather, they showed activity against *B. subtilis* and *S. aureus*, similar to *C. citratus* extracts. Although the *C. citratus* stem and root extracts only exhibited antibacterial activity against *B. subtilis* at 500 mg/mL, all *C. winterianus* extracts were effective at both 250 and 500 mg/mL. Additionally, only the stem extract of *C. winterianus* showed antibacterial activity against *S. aureus*, similar to *C. citratus*. However, its activity against *S. aureus* was weaker than that against *B. subtilis*. This result is consistent with that of Munda et al. [64] in which *C. winterianus* essential oil showed wider inhibition zones against *B. subtilis* than those against *S. aureus*. It also exhibited significant antibacterial activity against *E. coli* and antifungal activity against *C. albicans*, as demonstrated by wider inhibition zones; this contradicted our study findings. The percentage of the inhibition zones showed that the *C. nardus* root, stem, and leaf extracts were significantly effective antimicrobial agents, with the root extract showing the highest inhibition (127%).

*Cymbopogon* essential oil, also known as citronella oil, mainly consists of three compounds: citronellal, citronellol, and geraniol [65]. Citronellal is an aldehyde monoterpene with a fresh scent and desiccant characteristics. This compound causes cell death through dehydration. Citronellol and geraniol are alcoholic monoterpenes that inhibit bacterial growth by disrupting cell division or cellular membranes [66]. Kumala et al. [66] and Munda et al. [64] have demonstrated that *C. nardus* and *C. winterianus* essential oils contain high concentrations of these three compounds. Citral, another main component of these two essential oils, is a mixture of *trans*-isomers known as neural isomers [67]. Its content is used to determine the quality of essential oil. Moreover, high contents of this compound, geranial, and neral have been reported in *C. citratus* essential oil [68]. Citral is also abundant in the root stalk and shoot parts of *C. winterianus*. It inhibits bacterial growth by impairing cell membrane integrity, leading to membrane rupture [69]. Similarly, citronellal confers antifungal activity by inhibiting ergosterol biosynthesis, resulting in cell membrane damage [70].

The antibacterial and antifungal activities exhibited in the present study, especially by *C. nardus* extracts, were likely due to the abundance of these compounds. However, the abundance of these compounds was not the sole factor in their antimicrobial activity. Other factors, such as the resistance of gram-positive and gram-negative bacteria, may also play a role. The antimicrobial resistance of gram-positive bacteria is likely due to the presence of thick peptidoglycan in the cell wall, which makes it difficult for antimicrobial agents to penetrate. The antimicrobial resistance of gram-negative bacteria is due to porin channels in the outer membrane, where lipophilic drugs have difficulty penetrating [71]. This indicates that antagonistic and synergistic effects are caused by multiple components of the extracts [68].

In thi study, alpha-cadinol was predicted as a key compound in the mechanism underlying the antioxidant and antimicrobial activity of *Cymbopogon* spp. using in silico analysis (Fig. 6). In vitro antioxidant and antimicrobial assays also showed that *C. winterianus* and *C. nardus* leaf extracts had the best antioxidant and antimicrobial activities, with low cytotoxicity observed. Although *Cymbopogon* spp. generally demonstrated antioxidant and antimicrobial activities in the present study, *C. winterianus* and *C. nardus* leaf extracts were the most effective. These results suggest that *Cymbopogon* spp. extracts can be used to develop new antimicrobial drugs. However, bioassay-guided isolation is required to isolate pure compounds, such as alpha-cadinol.

## Conclusion

This study highlights the antioxidant and antimicrobial activities of *C. citratus* L., *C. nardus* (L.) Rendle., and *C. winterianus* Jowitt, as well as their low toxicity. We conclude that alpha-cadinol is a key *Cymbopogon* spp. compound that plays an important role in antioxidant and antibacterial activity pathways. *Cymbopogon* spp. leaves exhibited the highest antioxidant activity; *C. nardus* leaf extract exhibited the highest antimicrobial activity against *E. coli* and *S. aereus*; and *C. winterianus* stem extract showed the highest antimicrobial activity against *B. substilis*. These findings provide a theoretical basis for the clinical application of *Cymbopogon* spp.-derived compounds. However, bioassay-guided isolation of alpha-cadinol and clinical safety assessment of this extract and important compound are required for further pharmaceutical application.

### Supplementary Information

Additional file 1. Table of the yield of three commercial *Cymbopogon *spp. ethanolic extracts.

Additional file 2. Table of retention time and percentage area of metabolite profile of three commercial *Cymbopogon *spp. from Indonesia.

## Data Availability

All data generated or analysed during this study are included in this published article [and its supplementary information files].

## Abbreviations

GC-MS: gas chromatography-mass spectra
CID: cubic inch displacement
SDF: structure data format
PDB: protein data bank
ABTS: 2,2-azino-bis [3-ethylbenzothiazoline-6-sulfonic acid
DPPH: 2,2-diphenyl-1-picryl-hydrazyl-hydrate
NA: Nutrient Agar
PDA: Potato Dextrose Agar
NB: Nutrient Broth
DMSO: dimethyl sulfoxide
RMSF: Root Mean Square Fluctuation

## References

[1] WHO. World Health Organization. Global research agenda for antimicrobial resistance in human health. Geneva: WHO Press; 2023. p. 2023.

[2] Kementerian KRI, Kesehatan RI. Info DATIN Pusat Data dan Informasi Kesehatan Kementerian Kesehatan RI: Malaria. Jakarta: Kementerian Kesehatan Republik Indonesia; 2016. p. 2016.

[3] WHO. Meeting report,. Antimicrobial stewardship summit for selected member state in the WHO African Region. Adis Ababa, Ethiopia: World Health Organization; 2022. p. 2023.

[4] Sahal G, Woerdenbag HJ, Hinrichs WLJ, Visser A, Tepper PG, Quax WJ, et al. Antifungal and biofilm inhibitory effect of Cymbopogon citratus (lemongrass) essential oil on biofilm forming by Candida tropicalis isolates; an in vitro study. J Ethnopharmacol. 2020;246. 31470085 10.1016/j.jep.2019.112188

[5] Rahali FZ, Kefi S, Rebey IB, Hamdaoui G, Tabart J, Kevers C, et al. Phytochemical composition and antioxidant activities of different aerial parts extracts of Ferula communis L. Plant Biosyst. 2019;153:213–21. 10.1080/11263504.2018.1461696

[6] Baruah J, Gogoi B, Das K, Ahmed NM, Sarmah DK, Lal M, et al. Genetic diversity study amongst Cymbopogon species from NE-India using RAPD and ISSR markers. Ind Crops and Prod. 2017;95:235–43. 10.1016/j.indcrop.2016.10.022

[7] Fuentes-León F, González-Pumariega M, Tamayo MV, Menck CFM, Sánchez-Lamar A. Toxic evaluation of Cymbopogon citratus chemical fractions in E. coli. Cosmetics. 2017;4:20. 10.3390/cosmetics4020020

[8] Gao S, Liu G, Li J, Chen J, Li L, Li Z, et al. Antimicrobial activity of lemongrass essential oil (Cymbopogon flexuosus) and its active component citral against dual species biofilms of Staphylococcus aureus and Candida species. Front Cell Infect Microbiol. 2020;10. 33415085 10.3389/fcimb.2020.603858 PMC7783362

[9] Subramaniam G, Yew XY, Sivasamugham LA. Antibacterial activity of Cymbopogon citratus against clinically important bacteria. S Afr J Chem Eng. 2020;34:26–30.

[10] Zulfa Z, Chia CT, Rukayadi Y. In vitro antimicrobial activity of Cymbopogon citratus (lemongrass) extracts against selected foodborne pathogens. Int Food Res J. 2016;23:1262–7.

[11] Hasan ZYM, Al-Halbosiy MMF, Al-Lihaibi RK, Al-Nauimi EH. Short Communication: Antimicrobial of lemongrass (Cymbopogon citratus L.) volatile oil and cytotoxic effects against L20B and MCF-7cell lines. Biodiversitas. 2022;23:5298–301.

[12] Gaspar AL, Gaspar AB, Contini LRF, Silva MF, Chagas EGL, Bahú JO, et al. Lemongrass (Cymbopogon citratus)-incorporated chitosan bioactive films for potential skincare applications. Int J Pharm. 2022;628. 36270554 10.1016/j.ijpharm.2022.122301

[13] Kamal HZA, Ismail TNNT, Arief EM, Ponnuraj KT. Antimicrobial activities of citronella (Cymbopogon nardus) essential oil against several oral pathogens and its volatile compounds. Padjadjaran J Dent. 2020;32:1–7. 10.24198/pjd.vol32no1.24966

[14] Nurcholis W, Takene M, Puspita R, Tumanggor L, Qomaliyah EN, Sholeh MM. Antibacterial activity of lemongrass (Cymbopogon nardus) ethanolic extract. Curr Biochem. 2019;6:86–91.

[15] Hashim GM, Almasaudi SB, Azhar E, Al Jaouni SK, Harakeh S. Biological activity of Cymbopogon schoenanthus essential oil. Saudi J Biol Sci. 2017;24:1458–64. 30294213 10.1016/j.sjbs.2016.06.001 PMC6169510

[16] Brügger BP, Martínez LC, Plata-Rueda A, de Castro e Castro BM, Soares MA, Wilcken CF, et al. Bioactivity of the Cymbopogon citratus (Poaceae) essential oil and its terpenoid constituents on the predatory bug, Podisus nigrispinus (Heteroptera: Pentatomidae). Sci Rep. 2019;9(1):8358. 31175321 10.1038/s41598-019-44709-y PMC6555811

[17] Deletre E, Chandre F, Williams L, Duménil C, Menut C, Martin T. Electrophysiological and behavioral characterization of bioactive compounds of the Thymus vulgaris, Cymbopogon winterianus, Cuminum cyminum and Cinnamomum zeylanicum essential oils against Anopheles gambiae and prospects for their use as bednet treatments. Parasit Vectors. 2015;8:316. 26063119 10.1186/s13071-015-0934-y PMC4470088

[18] Anggraeni NI, Hidayat IW, Rachman SD, Ersanda. Bioactivity of essential oil from lemongrass (Cymbopogon citratus Stapf.) as antioxidant agent. AIP Conf Proc. 2018;1927:030007. 10.1063/1.5021200

[19] Hartatie ES, Prihartini I, Widodo W, Wahyudi A. Bioactive compounds of lemongrass (Cymbopogon citratus) essential oil from different parts of the plant and distillation methods as natural antioxidant in broiler meat. IOP Conf Ser: Mater Sci Eng. 2019;532:012018. 10.1088/1757-899X/532/1/012018

[20] Wińska K, Mączka W, Łyczko J, Grabarczyk M, Czubaszek A, Szumny A. Essential oils as antimicrobial agents–myth or real alternative? Molecules (Basel, Switzerland). 2019;24:2130. 31195752 10.3390/molecules24112130 PMC6612361

[21] Wahyuni DK, Wacharasindhu S, Bankeeree W, Wahyuningsih SPA, Ekasari W, Purnobasuki H, et al. n vitro and in vivo antiplasmodial activities of leaf extracts from Sonchus arvensis L. BMC Complement Med Ther. 2023;23(1):47. 36788545 10.1186/s12906-023-03871-7 PMC9926696

[22] Bressan EA, Rossi ML, Gerald LTS, Figueira A. Extraction of high-quality DNA from ethanol-preserved tropical plant tissues. BMC Res Notes. 2014;7:268. 24761774 10.1186/1756-0500-7-268 PMC4005624

[23] O’Boyle NM, Banck M, James CA, Morley C, Vandermeersch T, Hutchison GR. Open Babel: an open chemical toolbox. J Cheminform. 2011;3:33. 21982300 10.1186/1758-2946-3-33 PMC3198950

[24] Alotaibi BS. Targeting Filamenting temperature-sensitive mutant Z (FtsZ) with bioactive phytoconstituents: An emerging strategy for antibacterial therapy. PLoS ONE. 2023;18(8). 37647309 10.1371/journal.pone.0290852 PMC10468062

[25] Gan HX, Zhou H, Lin Q, Tong YW. Quantification of Aquaporin-Z reconstituted into vesicles for biomimetic membrane fabrication. Sci Rep. 2017;7:11565. 28912594 10.1038/s41598-017-11723-x PMC5599656

[26] Kumar A, White J, Christie RJ, Dimasi N, Gao C. Chapter Twelve – Antibody drug conjugates. Editor(s): Goodnow RA. Annual Reports in Medicinal Chemistry, Acad. Press. 2015;50:441–80.

[27] Pue N, Guddat LW. Acetohydroxyacid synthase: a target for antimicrobial drug discovery. Curr Pharm Des. 2014;20:740–53. 23688082 10.2174/13816128113199990009

[28] Abookleesh F, Mosa FES, Barakat K, Ullah A. Assessing molecular docking tools to guide the design of polymeric materials formulations: a case study of canola and soybean protein. Polymers (Basel). 2022;14:3690. 36080764 10.3390/polym14173690 PMC9460131

[29] Manjula R, Wright GSA, Strange RW, Padmanabhan B. Assessment of ligand binding at a site relevant to SOD1 oxidation and aggregation. FEBS Lett. 2018;592:1725–37. 29679384 10.1002/1873-3468.13055

[30] Ansori ANM, Kharisma VD, Parikesit AA, Dian FA, Probojati RT, Rebezov M, et al. Bioactive compounds from mangosteen (Garcinia mangostana L.) as an antiviral agent via dual inhibitor mechanism against SARS-CoV-2: an in silico approach. Pharmacogn J. 2022;14:85–90. 10.5530/pj.2022.14.12

[31] Dibha AF, Wahyuningsih S, Ansori ANM, Kharisma VD, Widyananda MH, Parikesit AA, et al. Utilization of secondary metabolites in algae Kappaphycus alvarezii as a breast cancer drug with a computational method. Pharmacogn J. 2022;14:536–43. 10.5530/pj.2022.14.68

[32] Amin MR, Yasmin F, Hosen MA, Dey S, Mahmud S, Saleh MA, et al. Synthesis, antimicrobial, anticancer, PASS, molecular docking, molecular dynamic simulations & pharmacokinetic predictions of some methyl β-D-galactopyranoside analogs. Molecules. 2021;26:7016. 34834107 10.3390/molecules26227016 PMC8621697

[33] Jianu C, Stoin D, Cocan I, David I, Pop G, Lukinich-Gruia AT, et al. In silico and in vitro evaluation of the antimicrobial and antioxidant potential of Mentha × smithiana R. GRAHAM essential oil from Western Romania. Foods. 2021;10:815. 33918674 10.3390/foods10040815 PMC8069324

[34] Mir WR, Bhat BA, Rather MA, Muzamil S, Almilaibary A, Alkhanani M, et al. A. Molecular docking analysis and evaluation of the antimicrobial properties of the constituents of Geranium wallichianum D. Don ex Sweet from Kashmir Himalaya. Sci Rep. 2022;12:12547. 35869098 10.1038/s41598-022-16102-9 PMC9307801

[35] Prieto JM. Procedure: preparation of DPPH radical, and antioxidant scavenging assay. In: Dr Prieto's DPPH Microplate Protocol. 2012. https://www.researchgate.net/-file.PostFileoader.html?id=503cd1c9e39d5ead11000043&assetKey=AS%3A271744332435456%401441800305338. Accessed 30 March 2021.

[36] Wahyuni DK, Wacharasindhu S, Bankeeree W, Punnapayak H, Prasongsuk S. In silico anti-SARS-CoV-2, antiplasmodial, antioxidant, and antimicrobial activities of crude extracts and homopterocarpin from heart wood of Pterocarpus macrocarpus Kurz. Heliyon. 2023;9:e13644. 36789389 10.1016/j.heliyon.2023.e13644 PMC9912040

[37] Wahyuni DK, Nariswari A, Supriyanto A, Purnobasuki H, Punnapayak H, Bankeeree W, et al. Antioxidant, antimicrobial, and antiplasmodial activities of Sonchus arvensis L. leaf ethyl acetate fractions. Pharmacogn J. 2022;14:993–8. 10.5530/pj.2022.14.202

[38] Khayer K, Haque T. Density functional theory calculation on the structural, electronic, and optical properties of fluorene-based azo compounds. ACS Omega. 2020;5:4507–31. 32175498 10.1021/acsomega.9b03839 PMC7066559

[39] Heintze AL, Schmidt D, Rodat T, Witt L, Ewert J, Kriegs M, et al. Photoswitchable azo- and diazocine-functionalized derivatives of the VEGFR-2 inhibitor axitinib. Int J Mol Sci. 2020;21:8961. 33255816 10.3390/ijms21238961 PMC7734574

[40] Volarić J, Buter J, Schulte AM, van den Berg K, Santamaría-Aranda E, Szymanski W, et al. Design and synthesis of visible-light-responsive azobenzene building blocks for chemical biology. J Org Chem. 2022;87:14319–33. 36285612 10.1021/acs.joc.2c01777 PMC9639001

[41] Mehdi MAH, Al-Alawi AMA, Thabet AZA, Alarabi FYS, Omar GMN, Pradhan V. Analysis of bioactive chemical compounds of leaves extracts from Tamarindus indica using FT-IR and GC-MS spectroscopy. Asian J Res Biochem. 2021;8:22–34. 10.9734/ajrb/2021/v8i130171

[42] Negreanu-Pirjol BS, Oprea OC, Negreanu-Pirjol T, Roncea FN, Prelipcean AM, Craciunescu O, et al. Health Benefits of Antioxidant Bioactive Compounds in the Fruits and Leaves of *Lonicera caerulea* L. and *Aronia melanocarpa* (Michx.) Elliot. Antioxidants. 2023;12:951. 37107325 10.3390/antiox12040951 PMC10136089

[43] Hassan A, Akmal Z, Khan N. The phytochemical screening and antioxidants potential of Schoenoplectus triqueter L. Palla J Chem. 2020;2020:3865139.

[44] Tong H, Wang X, Dong Y, Hu Q, Zhao Z, Zhu Y, et al. A Streptococcus aquaporin acts as peroxiporin for efflux of cellular hydrogen peroxide and alleviation of oxidative stress. J Biol Chem. 2019;294:4583–95. 30705089 10.1074/jbc.RA118.006877 PMC6433050

[45] Zhang D, Yang L, Su W, Zhao Y, Ma X, Zhou H, et al. Aquaporin-4 is downregulated in the basolateral membrane of ileum epithelial cells during enterotoxigenic Escherichia coli-induced diarrhea in mice. Front Microbiol. 2018;8:2655. 29375520 10.3389/fmicb.2017.02655 PMC5767235

[46] Thappeta KRV, Zhao LN, Nge CE, Crasta S, Leong CY, Ng V, et al. In-Silico Identified New Natural Sortase A Inhibitors Disrupt *S. aureus* Biofilm Formation. Int J Mol Sci. 2020;21:8601. 33202690 10.3390/ijms21228601 PMC7696255

[47] Garcia MD, Chua SMH, Low YS, Lee YT, Agnew-Francis K, Wang JG, et al. Commercial AHAS-inhibiting herbicides are promising drug leads for the treatment of human fungal pathogenic infections. Proc Natl Acad Sci USA. 2018;115:E9649–58. 30249642 10.1073/pnas.1809422115 PMC6187177

[48] Nandi A, Yan LJ, Jana CK, Das N. Role of catalase in oxidative stress- and age-associated degenerative diseases. Oxid Med Cell Longev. 2019;2019:9613090. 31827713 10.1155/2019/9613090 PMC6885225

[49] Sharma S, Singh S. Molecular docking study for binding affinity of 2H-thiopyrano[2,3-b]quinoline derivatives against CB1a. Interdiscip Perspect Infect Dis. 2023;2023:1618082. 36655217 10.1155/2023/1618082 PMC9842416

[50] Kharisma VD, Ansori ANM, Widyananda MH, Utami SL, Nugraha AP. Molecular simulation: the potency of conserved region on E6 HPV-16 as a binding target of black tea compounds against cervical cancer. Biochem Cell Arch. 2020;20(Suppl 1):2795–802.

[51] Muñoz M, Sánchez A, Martínez PM, Benedito S, López-Oliva ME, García-Sacristán A, et al. COX-2 is involved in vascular oxidative stress and endothelial dysfunction of renal interlobar arteries from obese Zucker rats. Free Radic Biol Med. 2015;84:77–90. 25841778 10.1016/j.freeradbiomed.2015.03.024

[52] Gonzalez-Paredes FJ, Mesa HG, Arraez MD, Reyes RM, Abrante B, Diaz-Flores F, et al. Contribution of cyclooxygenase end products and oxidative stress to intrahepatic endothelial dysfunction in early non-alcoholic fatty liver disease. PLoS ONE. 2016;11:e0156650. 27227672 10.1371/journal.pone.0156650 PMC4882009

[53] Herraiz T, Guillén H. Monoamine oxidase-A inhibition and associated antioxidant activity in plant extracts with potential antidepressant actions. Biomed Res Int. 2018;2018:4810394. 29568754 10.1155/2018/4810394 PMC5820675

[54] Mijatović S, Savić-Radojević A, Plješa-Ercegovac M, Simić T, Nicoletti F, Maksimović-Ivanić D. The double-faced role of nitric oxide and reactive oxygen species in solid tumors. Antioxidants (Basel). 2020;9:374. 32365852 10.3390/antiox9050374 PMC7278755

[55] Her L, Zhu HJ. Carboxylesterase 1 and precision pharmacotherapy: pharmacogenetics and nongenetic regulators. Drug Metab Dispos. 2020;48:230–44. 31871135 10.1124/dmd.119.089680 PMC7031766

[56] Unuigbe C, Enahoro J, Erharuyi O, Okeri HA. Phytochemical analysis and antioxidant evaluation of lemon grass (Cymbopogon citratus DC.) Stapf leaves. J Appl Sci Environ Manag. 2019;23:223–8.

[57] Rachmatillah A, Hasni D, Aisyah Y. Uji aktivitas antioksidan minyak sereh wangi (Cymbopogon nardus (L.) Rendle), minyak nilam (Pogostemson cablin Benth.) dan minyak pala (Myristica fragrans Houtt.). Jurnal Ilmiah Mahasiswa Pertanian. 2021;6:442–6. 10.17969/jimfp.v6i4.18245

[58] Shrestha D, Sharma P, Pandey A, Dhakal K, Baral RP, Adhikar A. Chemical characterization, antioxidant, and antibacterial activity of essential oil of Cymbopogon winterianus Jowitt (citronella) from Western Nepal. Curr Biotechnol. 2022;11:86–91. 10.2174/2211550111666220405133558

[59] Sahamastuti AAT, Andre A, Foustine S, Sumarpo A, Hartiadi LY. Synergistic antibacterial activities of ginger and lemongrass essential oils as an alternative prevention to food-borne disease. Indonesian J Life Sci. 2019;1:54–61. 10.54250/ijls.v1i2.25

[60] Pasquereau S, Nehme Z, Haidar Ahmad S, Daouad F, Van Assche J, Wallet C, et al. Resveratrol inhibits HCoV-229E and SARS-CoV-2 coronavirus replication in vitro. Viruses. 2021;13:354. 33672333 10.3390/v13020354 PMC7926471

[61] Pratama YM, Permana BP. Lemongrass (Cymbopogon citratus) essential oil inhibits Candida albicans growth in vitro. J Biomed. 2020;13:142–7.

[62] Yunita Y, Lestari F, Febrianti Y. Antibacterial activity lemongrass leaves of Staphylococcus aureus inhibition one. JPBIO. 2020;5:176–83.

[63] Shintawati S, Rina S, Ermaya D. Sifat antimikroba dan pengaruh perlakuan bahan baku terhadap rendeman minyak sereh wangi. Jurnal Sylva Lestari. 2020;8:411–9. 10.23960/jsl38411-419

[64] Munda S, Dutta S, Pandey SK, Sarma N, Lal M. Antimicrobial activity of essential oils of medicinal and aromatic plants of the North East India: A biodiversity hot spot. J Essent Oil-Bear Plants. 2019;22:105–19. 10.1080/0972060X.2019.1601032

[65] Sulaswatty A, Rusli MS, Abimanyu H, Tursiloadi S. Minyak serai wangi dan produk turunannya. Jakarta: LIPI Press; 2019.

[66] Kumala S, Anwar Y, Iftitah ED, Simanjuntak P. Isolasi dan identifikasi senyawa geraniol dari minyak atsiri tanaman sereh wangi Cymbopogon nardus (L) Rendle. Jurnal Ilmu Kefarmasian Indonesia. 2019;17:183–8. 10.35814/jifi.v17i2.746

[67] Kumoro AC, Wardhani DH, Retnowati DS, Haryani K. A brief review on the characteristics, extraction, and potential industrial applications of citronella grass (Cymbopogon nardus) and lemongrass (Cymbopogon citratus) essential oils. IOP Conf Ser: Mater Sci Eng. 2021;1053:012118. 10.1088/1757-899X/1053/1/012118

[68] Dangol S, Poudel DK, Ojha PK, Maharjan S, Poudel A, Satyal R, et al. Essential oil composition analysis of Cymbopogon species from Eastern Nepal by GC-MS and chiral GC-MS, and antimicrobial activity of some major compounds. Molecules. 2023;28:543. 36677603 10.3390/molecules28020543 PMC9863348

[69] Zhang Y, Wei J, Chen H, Song Z, Guo H, Yuan Y, et al. Antibacterial activity of essential oils against Stenotrophomonas maltophilia and the effect of citral on cell membrane. LWT. 2020;117:108667. 10.1016/j.lwt.2019.108667

[70] Yang OQ, Liu Y, Oketch OR, Zhang M, Shao X, Tao N. Citronellal exerts its antifungal activity by targeting ergosterol biosynthesis in Penicillium digitatum. J Fungi. 2021;7:432. 10.3390/jof7060432 PMC8229684 34072578

[71] Guimarães AC, Meireles LM, Lemos MF, Guimarães MCC, Endringer DC, Fronza M, et al. Antibacterial activity of terpenes and terpenoids present in essential oils. Molecules. 2019;24:2471. 31284397 10.3390/molecules24132471 PMC6651100

