# Correction: The antioxidant and antimicrobial activity of ethanolic extract in roots, stems, and leaves of three commercial *Cymbopogon* species

**DOI:** 10.1186/s12906-024-04599-8

**Published:** 2024-08-14

**Authors:** Dwi Kusuma Wahyuni, Viol Dhea Kharisma, Ahmad Affan Ali Murtadlo, Cici Tya Rahmawati, Alvi Jauharotus Syukriya, Sehanat Prasongsuk, Sreeramanan Subramaniam, Anjar Tri Wibowo, Hery Purnobasuki

**Affiliations:** 1 Department of Biology, Faculty of Science and Technology, Universitas Airlangga Surabaya, East Java, 60115 Indonesia; 2 Program in Biotechnology, Faculty of Science, Chulalongkorn University, Bangkok, 10330 Thailand; 3 Plant Biomass Utilization Research Unit, Department of Botany, Faculty of Science, Chulalongkorn University, Bangkok, 10330 Thailand; 4 School of Biological Science, Universiti Sains Malaysia, Georgetown, 11800 Malaysia

**Correction: BMC Complement Med Ther 24**,** 272 (2024)**

10.1186/s12906-024-04573-4

Following publication of the original article [1], the authors identified an error in Fig. 1. The correct figure is given below.

**Fig. 1:**
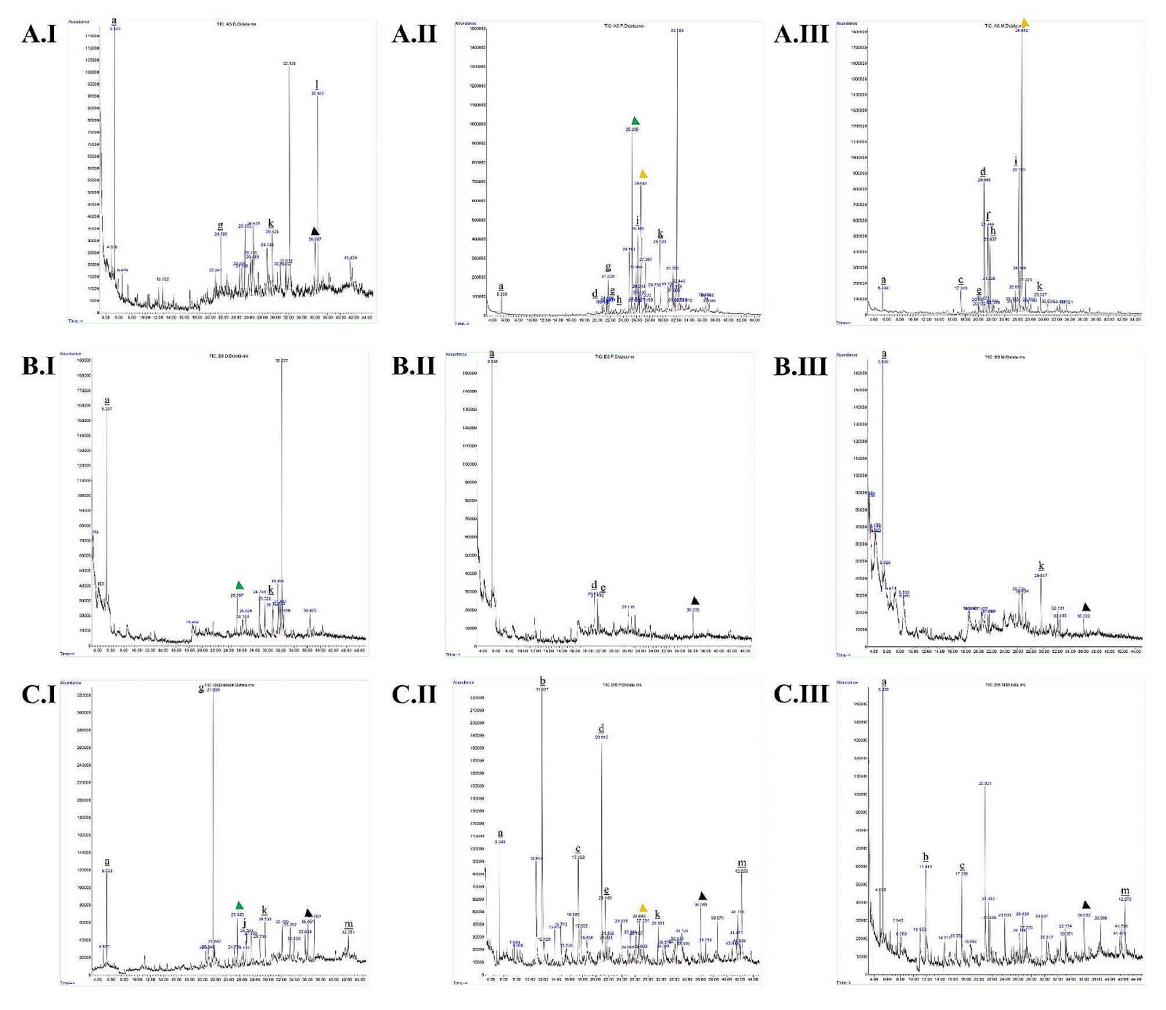
GC–MS chromatogram of *Cymbopogon spp*. ethanolic extract. A Roots, B stems, C leaves. (I) Cymbopogon citratus, (II) Cymbopogon nardus, III Cymbopogon winterianus. (**a**) Tetraethyl silicate; (**b**) Geraniol; (**c**) Methyleugenol; (**d**) Benzene, 1,2-dimethoxy-4-(1-propenyl)-; (**e**) Naphthalene, 1,2,3,4,4a,5,6,8a-octahydro-7-methyl-4-methylene-1-(1-methylethyl)-, (1.alpha.,4a.beta.,8a.alpha.)-; f. gamma.-Muurolene; g. Phenol, 2,5-bis(1,1-dimethylethyl); h. Naphthalene, 1,2,3,5,6,8a-hexahydro-4,7-dimethyl-1-(1-methylethyl)-, (1 S-cis)-; i. tau.-Muurolol; j. (1 S,4aS,7R,8aS)-1,4a-Dimethyl-7-(prop-1-en-2-yl) decahydronaphthalen-1-ol; k. 1-((1 S,3aR,4R,7 S,7aS)-4-Hydroxy-7-isopropyl-4-methyloctahydro-1 H-inden-1-yl) ethanone; l. Benzenepropanoic acid, 3,5-bis (1,1-dimethylethyl)-4-hydroxy-, methyl ester; m. Phytol. Green arrow: Selin-6-en-4.alpha.-ol; yellow arrow: alpha.-Cadinol; black arrow: Hexadecanoic acid, methyl ester

The original article has been corrected.

## References

[1] Wahyuni DK, Kharisma VD, Murtadlo AAA, et al. The antioxidant and antimicrobial activity of ethanolic extract in roots, stems, and leaves of three commercial *Cymbopogon* species. BMC Complement Med Ther. 2024;24:272. 10.1186/s12906-024-04573-4. 39026301 10.1186/s12906-024-04573-4 PMC11264733

