# Supplementary material for: The antioxidant and antimicrobial activity of ethanolic extract in roots, stems, and leaves of three commercial Cymbopogon species
Source: BMC Complement Med Ther. 2024 Jul 18;24:272. doi: 10.1186/s12906-024-04573-4 (PMC11264733; doi:10.1186/s12906-024-04573-4)
Supplement: Supplementary file 1 — Additional file 1. Table of the yield of three commercial Cymbopogon spp. ethanolic extracts. [file 12906_2024_4573_MOESM1_ESM.docx]

**Additional file 1.** Table of the yield of three commercial *Cymbopogon* spp. ethanolic extracts

| Species | The weight of raw material (gram) | | | | The weight of extract (gram) | | | The yield of extract (%) | | | |
| --- | --- | --- | --- | --- | --- | --- | --- | --- | --- | --- | --- |
|  | leaves | stems | roots | leaves | | stems | roots | leaves | stems | roots |  |
| *C. citratus* | 10±0.00 | 10±0.00 | 10±0.00 | 1.14±0.01 | | 1.36±0.03 | 1.46±0.05 | 11.40±0.12 | 13.62±0.26 | 14.61±0.46 |  |
| *C. nardus* | 10±0.00 | 10±0.00 | 10±0.00 | 1.23±0.05 | | 1.53±0.30 | 1.78±0.06 | 12.36±0.49 | 15.26±0.36 | 17.76±0-62 |  |
| *C. wintherianus* | 10±0.00 | 10±0.00 | 10±0.00 | 1.36±0.08 | | 1.26±0.03 | 1.41±0.04 | 13.56±0.75 | 12.56±0.22 | 14.11±0.40 |  |
