# Supplementary material for: The antioxidant and antimicrobial activity of ethanolic extract in roots, stems, and leaves of three commercial Cymbopogon species
Source: BMC Complement Med Ther. 2024 Jul 18;24:272. doi: 10.1186/s12906-024-04573-4 (PMC11264733; doi:10.1186/s12906-024-04573-4)
Supplement: Supplementary file 2 — Additional file 2. Table of retention time and percentage area of metabolite profile of three commercial Cymbopogon spp. from Indonesia. [file 12906_2024_4573_MOESM2_ESM.docx]

**Additional file 2.** Table of retention time and percentage area of metabolite profile of three commercial *Cymbopogon* spp. from Indonesia

| **No** | **Compound** | **RT**  **(min)** | **Relative area peak (%)** | | | | | | | | |
| --- | --- | --- | --- | --- | --- | --- | --- | --- | --- | --- | --- |
|  |  |  | **CNL** | **CWL** | **CCL** | **CNS** | **CWS** | **CCS** | **CNR** | **CWR** | **CCR** |
| 1 | 1-Cyclohexylnonene | 3.296 |  |  |  |  |  | 4.07 |  |  |  |
| 2 | Disiloxane, 1,3-diethoxy-1,1,3,3-tetramethyl- | 4.875 |  | 1.81 | 0.89 |  |  |  |  |  |  |
| 3 | Tetraethyl silicate | 5.337 | 1.78 | 6.24 | 3.73 | 37.17 | 15.53 | 9.11 | 0.42 | 0.64 | 6.33 |
| 4 | 2H-Pyran, tetrahydro-4-methyl-2-(2-methyl-1-propenyl)- trans-Rose oxide | 7.549 | 0.49 | 1.31 |  |  |  |  |  |  |  |
| 5 | 2-Octen-1-ol, 3,7-dimethyl- | 10.947 | 6.24 | 2.09 |  |  |  |  |  |  |  |
| 6 | Geraniol | 11.834 | 17.15 | 11.27 |  |  |  |  |  |  |  |
| 7 | (1S,2R,5R)-2-(2-Hydroxypropan-2-yl)-5-methylcyclohexanol | 14.713 | 2.71 | 2.32 |  |  |  |  |  |  |  |
| 8 | (1S,2R,5R)-2-(2-Hydroxypropan-2-yl)-5-methylcyclohexanol | 15.530 | 0.91 |  |  |  |  |  |  |  |  |
| 9 | Geranyl acetate | 16.549 | 1.73 | 1.62 |  |  |  |  |  |  |  |
| 10 | Methyleugenol | 17.352 | 5.76 | 8.87 |  |  |  |  |  | 2.34 |  |
| 11 | Caryophyllene | 17.835 | 1.27 |  |  |  |  |  |  |  |  |
| 12 | (3R,3aR,3bR,4S,7R,7aR)-4-Isopropyl-3,7-dimethyloctahydro-1H-cyclopenta[1,3]cyclopropa[1,2]benzen-3-ol | 20.721 |  |  |  |  |  |  |  | 1.00 |  |
| 13 | Benzene, 1,2-dimethoxy-4-(1-propenyl)- | 20.915 | 13.11 | 16.59 |  | 20.56 |  |  | 0.80 | 13.32 |  |
| 14 | Germacrene D | 20.804 |  |  |  |  | 2.86 |  |  | 0.57 |  |
| 15 | Naphthalene, 1,2,3,4,4a,5,6,8a-octahydro-7-methyl-4-methylene-1-(1-methylethyl)-, (1.alpha.,4a.beta.,8a.alpha.)- | 21.086 | 2.73 |  |  | 15.24 |  |  | 0.63 | 0.90 |  |
| 16 | Bicyclo[4.4.0]dec-1-ene, 2-isopropyl-5-methyl-9-methylene- | 21.442 |  | 4.01 |  |  |  |  |  |  |  |
| 17 | .gamma.-Muurolene | 21.444 |  |  |  |  |  |  |  | 7.79 |  |
| 18 | (3S,3aR,3bR,4S,7R,7aR)-4-Isopropyl-3,7-dimethyloctahydro-1H-cyclopenta[1,3]cyclopropa[1,2]benzen-3-ol | 21.508 |  |  |  |  |  |  | 0.75 |  |  |
| 19 | 2,4-Di-tert-butylphenol | 21.628 |  |  |  |  |  |  |  | 2.91 |  |
| 20 | Phenol, 2,5-bis(1,1-dimethylethyl) | 21.627 |  |  | 27.87 |  |  |  | 2.10 |  | 3.75 |
| 21 | 1-(3,6,6-Trimethyl-1,6,7,7a-tetrahydrocyclopenta[c]pyran-1-yl)ethanone | 21.633 | 0.81 |  |  |  |  |  |  |  |  |
| 22 | Naphthalene, 1,2,3,5,6,8a-hexahydro-4,7-dimethyl-1-(1-methylethyl)-, (1S-cis)- | 21.806 |  |  |  |  |  |  | 0.77 | 6.45 |  |
| 23 | cis-muurola-3,5-diene | 22.148 |  |  |  |  |  |  |  | 0.97 |  |
| 24 | Naphthalene, 1,2,4a,5,6,8a-hexahydro-4,7-dimethyl-1-(1-methylethyl)- , [1S-(1.alpha.,4a.beta.,8a.alpha. )]- | 22.323 |  |  |  |  |  |  |  | 0.60 |  |
| 25 | .alpha.-epi-7-epi-5-Eudesmol | 24.866 |  |  | 2.31 |  |  |  | 4.03 |  |  |
| 26 | Epicubenol | 25.183 |  |  |  |  |  |  |  | 1.62 |  |
| 27 | Azulene, 1,2,3,3a,4,5,6,7-octahydro-1,4-dimethyl-7-(1-methylethenyl) -, [1R-(1.alpha.,3a.beta.,4.alpha. ,7.beta.)]- | 25.299 | 1.78 |  |  |  |  |  |  |  |  |
| 28 | Selin-6-en-4.alpha.-ol | 25.302 |  |  | 6.66 |  |  | 3.97 | 12.46 |  |  |
| 29 | 1H-Cycloprop[e]azulene, 1a,2,3,4,4a,5,6,7b-octahydro-1,1,4,7-tetramethyl-, [1aR-(1a.alpha.,4.alpha.,4a.beta.,7b.alpha.)]- | 25.303 |  |  |  |  |  |  |  |  | 5.32 |
| 30 | 2,6,10-Dodecatrien-1-ol, 3,7,11-trimethyl-, (Z,E)- | 25.344 |  |  |  |  |  |  |  | 0.85 |  |
| 31 | Muurola-4,10(14)-dien-1.beta.-ol | 25.672 |  |  |  |  |  |  |  | 1.92 |  |
| 32 | .gamma.-Muurolene | 25.680 |  |  |  |  |  |  | 0.89 |  |  |
| 33 | .tau.-Muurolol | 26.187 |  |  |  |  |  |  | 6.97 | 18.33 |  |
| 34 | Copaene | 26.344 |  |  |  |  |  |  | 1.49 | 3.78 |  |
| 35 | 1-Isopropyl-4,7-dimethyl-1,2,3,4,5,6-hexahydronaphthalene | 26.157 | 2.09 |  |  |  |  |  |  |  |  |
| 36 | 2-Naphthalenemethanol, decahydro-.alpha.,.alpha.,4a-trimethyl-8-methylene-, [2R-(2.alpha.,4a.alpha.,8a.beta.)]- | 26.546 |  |  |  |  |  |  | 1.12 |  |  |
| 37 | .alpha.-Cadinol | 26.639 | 2.87 |  |  |  |  |  | 14.23 | 26.76 |  |
| 38 | (1S,4aS,7R,8aS)-1,4a-Dimethyl-7-(prop-1-en-2-yl)decahydronaphthalen-1-ol | 26.703 |  |  | 8.38 |  |  |  |  |  |  |
| 39 | (1R,7S,E)-7-Isopropyl-4,10-dimethylenecyclodec-5-enol | 27.742 |  |  |  |  |  |  |  | 1.10 |  |
| 40 | 6,10-Dodecadien-1-ol, 3,7,11-trimethyl- | 28.032 |  |  |  |  |  |  |  | 0.94 |  |
| 41 | 1-((1S,3aR,4R,7S,7aS)-4-Hydroxy-7-isopropyl-4-methyloctahydro-1H-inden-1-yl)ethanone | 29.526 | 1.90 |  | 5.65 |  | 8.56 | 4.69 | 4.76 | 1.56 | 4.79 |
| 42 | 7-Acetyl-2-hydroxy-2-methyl-5-isopropylbicyclo[4.3.0]nonane | 29.537 |  | 4.13 |  |  |  |  |  |  |  |
| 43 | 6-Hydroxy-4,4,7a-trimethyl-5,6,7,7a-tetrahydrobenzofuran-2(4H)-one | 30.575 | 1.91 |  |  |  |  |  |  |  |  |
| 44 | -(2-Hydroxypropan-2-yl)-1,4a-dimethyldecahydronaphthalen-1-ol | 30.756 |  |  |  |  |  |  | 2.82 |  |  |
| 45 | 2-Pentadecanone, 6,10,14-trimethyl | 33.352 |  |  | 3.90 |  |  |  |  |  |  |
| 46 | 9-Isopropyl-1-methyl-2-methylene-5-oxatricyclo[5.4.0.0(3,8)]undecane | 33.512 |  |  |  |  |  |  | 0.57 |  |  |
| 47 | 7,9-Di-tert-butyl-1-oxaspiro(4,5)deca-6,9-diene-2,8-dione | 35.654 |  |  | 3.44 |  |  |  |  |  |  |
| 48 | Hexadecanoic acid, methyl ester | 36.044 | 2.86 | 2.93 | 3.64 | 9.99 | 1.99 |  |  |  | 4.92 |
| 49 | Benzenepropanoic acid, 3,5-bis(1,1-dimethylethyl)-4-hydroxy-, methyl ester | 36.457 |  |  |  |  |  |  |  |  | 13.50 |
| 50 | 1,2-Benzenedicarboxylic acid, butyl decyl ester | 37.053 |  |  | 4.83 |  |  |  |  |  |  |
| 51 | Methyl 7,12-octadecadienoate | 41.472 | 1.60 | 0.97 |  |  |  |  |  |  | 2.13 |
| 52 | 9,12,15-Octadecatrienoic acid, methyl ester, (Z,Z,Z)- | 41.718 | 3.01 | 3.17 |  |  |  |  |  |  |  |
| 53 | Phytol | 42.261 | 6.46 | 5.72 | 2.50 |  |  |  |  |  |  |

Note: RT: retention time; CNL: *Cymbopogon nardus* leaves; CWL: *Cymbopogon winterianus* leaves; CCL: *Cymbopogon citratus* leaves; CNS: *Cymbopogon nardus* stems; CWS: *Cymbopogon winterianus* stems; CCS: *Cymbopogon citratus* stems; CNR: *Cymbopogon nardus* roots; CWR: *Cymbopogon winterianus* roots; CCR: *Cymbopogon citratus* roots.
